# Single‐cell transcriptome sequencing of B‐cell heterogeneity and tertiary lymphoid structure predicts breast cancer prognosis and neoadjuvant therapy efficacy

**DOI:** 10.1002/ctm2.1346

**Published:** 2023-08-01

**Authors:** Qing Wang, Ke Sun, Rui Liu, Ying Song, Yafeng Lv, Pingping Bi, Fuying Yang, Sijia Li, Jiawen Zhao, Xiuqin Li, Dong Chen, Jialin Mei, Rirong Yang, Kai Chen, Dequan Liu, Shichong Tang

**Affiliations:** ^1^ Department of Breast Surgery Caner Hospital of Yunnan Province The Third Affiliated Hospital of Kunming Medical University Kunming China; ^2^ State Key Laboratory of Primate Biomedical Research Institute of Primate Translational Medicine Kunming University of Science and Technology Kunming China; ^3^ Yunnan Key Laboratory of Primate Biomedical Research Kunming China; ^4^ Department of Ultrasound Caner Hospital of Yunnan Province The Third Affiliated Hospital of Kunming Medical University Kunming China; ^5^ Department of Cardiothoracic Surgery Baoshan People's Hospital Baoshan China; ^6^ Center for Genomic and Personalized Medicine Guangxi Medical University Nanning China; ^7^ Department of Immunology School of Basic Medical Sciences Guangxi Medical University Nanning China

**Keywords:** B cells, breast cancer, single cell sequencing, tertiary lymphoid structures, treatment

## Abstract

**Background:**

Breast cancer (BC) is a highly heterogeneous disease, and although immunotherapy has recently increased patient survival in a number of solid and hematologic malignancies, most BC subtypes respond poorly to immune checkpoint blockade therapy (ICB). B cells, particularly those that congregate in tertiary lymphoid structures (TLS), play a significant role in antitumour immunity. However, B‐cell heterogeneity at single‐cell resolution and its clinical significance with TLS in BC need to be explored further.

**Methods:**

Primary tumour lesions and surrounding normal tissues were taken from 14 BC patients, totaling 124,587 cells, for single‐cell transcriptome sequencing and bioinformatics analysis.

**Results:**

Based on the usual markers, the single‐cell transcriptome profiles were classified into various clusters. A thorough single‐cell study was conducted with a focus on tumour‐infiltrating B cells (TIL‐B) and tumour‐associated neutrophils (TAN). TIL‐B was divided into five clusters, and unusual cell types, such as follicular B cells, which are strongly related to immunotherapy efficacy, were identified. In BC, TAN and TIL‐B infiltration are positively correlated, and at the same time, compared with TLS‐high, TAN and TIL‐B in TLS‐low group are significantly positively correlated.

**Conclusions:**

In conclusion, our study highlights the heterogeneity of B cells in BC, explains how B cells and TLS contribute significantly to antitumour immunity at both the single‐cell and clinical level, and offers a straightforward marker for TLS called CD23. These results will offer more pertinent information on the applicability and effectiveness of tumour immunotherapy for BC.

## Graphical abstract

- Single cell RNA‐seq reveals the effect of TLS on tumour microenvironment.
- TLS‐negative group showed obvious immunosuppressive environment and pro‐tumour metabolic pathways.
- CD23 is a potential biomarker of TLS.
- TLS is associated with better treatment response and prognosis.

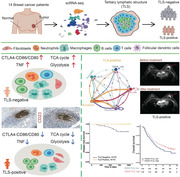

## 1 BACKGROUND

Breast cancer (BC) is a highly heterogeneous disease that can be classified into a number of subtypes with various biological characteristics and histological subtypes, each of which has a distinct clinical behavior and treatment sensitivity.^1^ Single‐cell RNA sequencing (scRNA‐seq) has transformed how we comprehend cancers and provided new information on their cellular makeup.^2^ In these studies, cell types are subdivided into finer subclassifications, which form an extremely complex tumour microenvironment (TME) that constantly interacts with itself and the tumour to coregulate it, but many of the underlying mechanisms relating to the roles of tumour infiltration‐associated cell subpopulations and how they interact remain unexplored.

The TME is a biological milieu composed of immune cells, blood vessels, extracellular matrix, fibroblasts, and lymphocytes that surrounds tumour or cancer stem cells, which is crucial to the growth, therapy, and prognosis of cancer.^3^ T lymphocyte‐mediated adaptive cellular immunity has been shown to play an important role in tumour immune responses,^4^ but the impact of B lymphocytes on tumours has only recently been recognized,^5^ and numerous studies have shown that tumour‐infiltrating B cells (TIL‐B) are associated with the patient's prognosis.^6^ In particular, the role of B‐cell subsets in BC and their underlying mechanisms are still under debate.^7^

Tertiary lymphoid structures (TLS) are ectopic lymphoid organs that form in nonlymphoid tissues at locations of persistent inflammation (including malignancies), and mature TLSs are distinguished by the presence of B‐cell‐rich regions.^8^ There is increasing evidence that, in the majority of solid tumour types, the presence of TLS is substantially correlated with a decreased risk of recurrence and increased effectiveness of immune checkpoint blockade (ICB).^8^ While ICB has recently improved patient survival in a number of solid and hematologic malignancies, it has little effect on the majority of BC subtypes and is currently only approved for combination therapy in programmed cell death‐ligand 1 (PD‐L1)‐positive triple‐negative BC (TNBC).^9^ A recent study used a 9‐gene TLS signature to assess the presence of TLSs in BC and found a positive correlation between the presence of TLSs and early tumour TNM stage, as well as a better prognosis for BC patients with high TLS.^10^ However, the methods used to assess TLS in that study are not suitable for use in routine diagnosis.

Recent research has shown that TAN can promote the recruitment of B cells and aid in their differentiation into plasma cells, but this finding is primarily applicable to lung cancer.^11^ The effect of an interaction between TAN and B cells in BC is still unknown. Some research evidence now supports the concept that TAN exhibits functional flexibility driven by numerous variables present in the TME.^12^, ^13^ There is currently no research analyzing the alterations and molecular mechanism of TAN in TME with TLS.

In this study, we applied scRNA‐seq to analyze human BC to understand the various functions and interactions of tumour‐infiltrating immune cells, particularly TIL‐B and TAN, as well as to elaborate on the clinical relevance of TLS to chemotherapy and immunotherapy and to search for straightforward TLS biomarkers to understand more fully the function and prognostic relevance of TLS.

## 2 METHODS

All of the BC patients who took part in the scRNA‐seq analysis signed a written informed consent form stating that their tumour materials and data would be used in this study. The ethics committee of Kunming Medical University's Third Affiliated Hospital approved the research protocol.

### 2.1 Patient material

**FIGURE 1:**
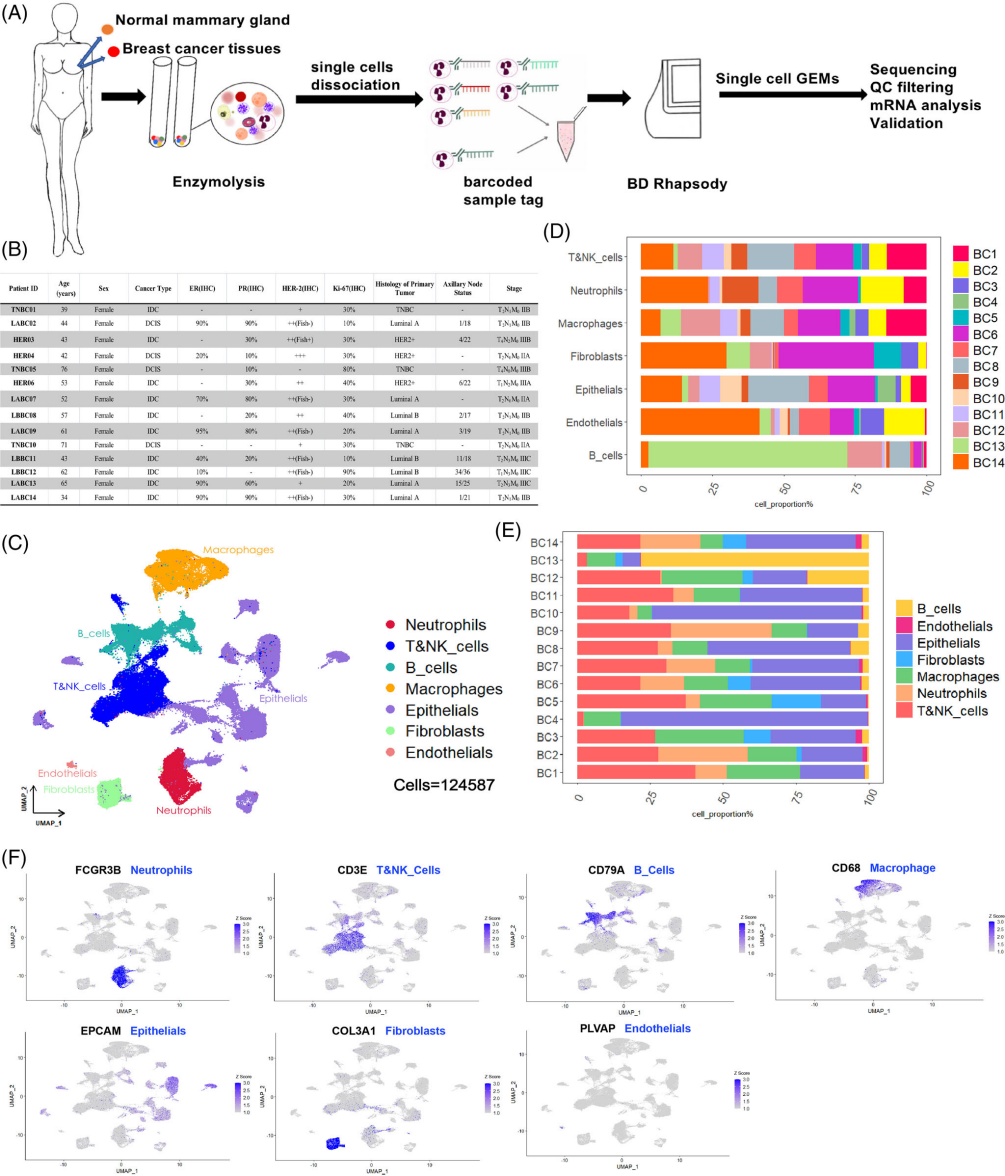
Single cell transcriptome data integration to identify the tumour microenvironment (TME) in breast cancer (BC). (A) The workflow diagram gives a general overview of the procedures involved in gathering and preparing samples for scRNA‐seq analysis. (B) Clinical data from breast cancer patients enrolled in the trial. (C) In this study, the cell types of scRNA‐seq data were displayed using the uniform manifold approximation and projection (UMAP) projection (124 587 cells; 14 patients). (D) Percent of each type of cell in samples of breast cancer. (E) Distribution of major cell types in each breast cancer sample. (F) The exemplary typical marker genes and their uniform expression levels in each cluster are displayed on the featureplot.

The tumour materials used in this investigation were from the surgical wastes of patients treated at Kunming Medical University's Third Affiliated Hospital. The malignant tumour and nearby tissues were obtained for scRNA‐seq detection after BC was detected by pathological biopsy. There were no other selection criteria utilized throughout the sample collection, and no cases were chosen based on the presence of TLS. Finally, 14 patients were enrolled in the study (2020–2021), all of them were female and had an average age of 52.5 years, with 10 patients having IDC and four having DCIS. All patients had not had chemotherapy or radiotherapy prior to surgical treatment, and all acquired materials were processed and stored anonymously. Their clinical characteristics are summarized in Figure 1B.

### 2.2 Single‐cell RNA sequencing

The freshly obtained tumor tissue was placed on ice and then finely diced into small fragments (approximately 1 mm^3^) using sterile scissors. Subsequently, the tissue was thoroughly mixed with Hanks balanced salt solution (HBSS) (02‐018‐1A, biological industries), 1.5 mg/µL of type collagenase (40C20079, Worthington), 1.5 mg/µL of type IV collagenase (40N20647, Worthington), and 5 U/L of recombinant human DNase (10104159001, Sigma‐Aldrich). After digestion at 37°C for 10 minutes, the process was completed. Filter digests with 40‐μm sterile filter (Falcon), and centrifuge the filtered cell suspension at 400 × g for 5 min to remove the supernatant. To lyse the red blood cells, suspend the cells in 10 mL ACK Lysis Buffer (abs9101, Absin), add 1‐mL Red Blood Cell Lysis Buffer (64010‐00‐100, BioGems) and incubate on ice for 7 min.

The supernatant was removed, and the cells were resuspended in 1‐mL FBS (AB 2869006, BD) after centrifugation at 400 × g for 5 min. The number and vitality of cells were evaluated before entering the BD RhapsodyTM platform using Countess 3 automatic cell counter (Invitrogen), and the end survival rate was >85%.

The single cell library was created using the BD Rhapsody WTA Kit (633802; BD Biosciences) according to the BD Rhapsody Protocol (633701; BD Biosciences, CA, USA).^14^ After the cell suspension was multi‐labeled with a 45‐nucleotide barcode label, it was randomly loaded into BD Rhapsody Cartridge with over 200 000 micropores, and cell capture magnetic beads with a unique molecular identifier (UMI) were added in excess to the cartridge. The cells were then lysed, the RNA molecules were mixed with magnetic beads, and reverse transcription, amplification and purification were performed. Finally, the cDNA library and Sample Tag library were prepared using the BD Rhapsody WTA Kit (633802; BD Biosciences). The Illumina Novaseq device was used to sequence the data in PE150 mode.

### 2.3 Pretreatment of scRNA‐seq data

The sequencing data were processed using the Seven Bridges (https://www.sevenbridges.com) standard BD Rhapsody Whole Transcriptome Assay Analysis Pipeline, which included quality control, filtration, annotation, and the development of gene expression profiles. After generating FASTQ files with NovaSeq, the CellRanger toolkit (V2.1.1) was used to compare the single cell expression matrix of each sample to the human reference genome (HG38). For quality control and downstream analysis, single‐cell sequencing data were analyzed using R software (Seurat package [V4.0.6]). Low‐quality cells with less than 300 genes or more than 10% mitochondrial genes were filtered initially, then double cells were deleted using the DoubletFinder program (V2.0.2).^15^ Finally, for the next study, 10,445 cells were employed.

### 2.4 Processing and annotation of scRNA‐seq

For analysis annotation and visualization of pre‐processed single‐cell sequencing data, Seurat was used to NormalizeData, ScaleData, reduce batch effect, and FindClusters with resolution of 1.9. Each cell cluster was visualized using uniform manifold approximation and projection (UMAP), and variable genes were found using FindVariableGenes. To find gene expression markers in different cell clusters, Seurat FindAllMarkers was employed. To acquire more detailed annotations, B cells and neutrophils were separated, and t‐SNE(RunTSNE), FindNeighbors and FindClusters were recalculated. The IGLC, IGKC and IGHG, IGHA clusters are visualized using UMAP and FeaturePlots. Using Single Sample Gene Set Enrichment Analysis (ssGSEA) to clarify the identity of each B‐cell subpopulation.

### 2.5 Determination of DEGs and GO enrichment analysis

FindAllMarkers in the Seurat package was used to locate genes that were differentially overexpressed in distinct clusters in standardized B cells and neutrophils. *p*‐Values less than .05 were considered statistically significant and were used in the gene ontology (GO) enrichment analysis. For clustering specific biomarker gene enrichment analysis, the ClusterProfiler program (V3.14.3) and the GOplot package's GoChord function were utilized. From the molecular signature Database (MSigDB), we used GSEA (V4.0.3) with representative gene sets to find pathways that were activated or suppressed between cell clusters.

### 2.6 Pseudo‐temporal trajectory analysis was used to infer the development of TIL‐B

The pseudo‐time locus was created with the Monocle 2 package in R (V2.8.0), which was used to extract all B‐cell clusters from scRNA‐seq data of BC patients and feed the gene‐cell matrix from the original UMI count scale of these cells into Monocle 2.

To make the Monocle 2 object, use the new CellDataSet function. The variable genes chosen by Seurat are utilized to reduce the reducedimension function's dimension, and plot‐cell‐Trajectory was used to sort and illustrate TIL‐B. The genes that change with the pseudo‐time of cell development (q ‐val < 10 −10) are calculated, and the plot‐pseudotime‐heatmap function is used to visualize them. The clusterProfiler package (V3.18.0) was used to divide these pseudo time‐dependent genes into subgroups based on their gene expression patterns and to perform GO enrichment analysis.

### 2.7 Analysis of cell‐to‐cell communication

The intercellular ligand‐receptor interactions were investigated using CellphoneDB (www.cellphonedb.org) and CellChat to better understand the potential interactions between TLS‐positive and TLS‐negative in BC. CellPhoneDB is a database of receptors, ligands, and their interactions that is open to the public. To calculate the *p* value of the specific cell type possibility of the matching receptor‐ligand complex, we use the default ligand‐receptor interaction data and 1000 permutation tests to determine the correlation between cell receptors and ligands. Then we choose the biologically related ligand‐receptor pair with the *p* value less than .05.

By analyzing the expression of known ligand‐receptor pairs, we use CellChat (V1.1.3) to infer the communication network between immune cells in BC. We also choose ‘Secret Signaling’ and ‘cell‐cell contact’ pairing data for cell communication analysis, compare the number and weight of outgoing and incoming interactions of each pair of cells, and use netVisual heatmap, netVisual bubble and circle plot in CellChat to visualize the differences in immune communication networks.

### 2.8 Gene expression analysis of FCER2 (CD23)

Studying FCER2 (CD23) in various tumour types of tumour and non‐tumour tissues is done using the ‘Gene DE’ module of the Tumor Immune Estimation Resource version 2 (TIMER2) (http://timer.cistrome.org/). To investigate the relationship between CD23 expression and major immune cell infiltration, researchers used TIMER2's ‘Immunity’ module, Extended Multidimensional Immunome Characterization (EPIC) and Tumor Immune Dysfunction and Exclusion (TIDE) algorithms.

### 2.9 Survival analysis

The ‘survival analysis’ module of Gene Expression Profile Interaction Analysis version 2 (GEPIA2) (http://gepia2. cancer‐pku.cn/) was used to construct Kaplan–Meier plots of CD69, FCER2 and apolipoprotein D (APOD) in The Cancer Genome Atlas (TCGA) Breast Cancer Aggressive Carcinoma (BRCA) cohort.

Download RNA‐seq gene expression profile and clinical information of BRCA from TCGA database (https://gdc‐portal.nci.nih.gov/). After matching with Propensity score matching (PSM), survival analysis and prognostic factor analysis were performed. For survival analysis, the Kaplan–Meier method and the log‐rank test were employed. For prognostic factors, COX proportional hazard regression model was used. The *p* values were calculated using the two‐sided log rank test. The R package survival (V3.40‐0) was utilized for the survival analysis and HR calculation.

### 2.10 Investigating the single‐cell metabolic properties of immune cells in BC

Single‐cell metabolism can be measured using the R package scMetabolism. We gathered single‐cell matrix data of immune cells from 14 BC patients in order to examine the metabolic characteristics of tumour‐infiltrating immune cells at the single‐cell level. Then, we employed a visual algorithm to score each cell using the scMetabolism program. The average metabolic gene expression heat map of immune cells in BC was also mapped using the Kyoto encyclopedia of genes and genomes (KEGG) database.

### 2.11 BRCA TCGA data DEGs analysis

Downloaded the TLS H&E images, along with the relevant clinical and RNA‐seq data, from https://portal.gdc.cancer.gov for a comprehensive assessment of the TCGA cohort in BRCA, aiming to further evaluate Tertiary Lymphoid Structure (TLS). The differentially expressed genes (DEGs) in TLS‐positive and TLS‐negative BC patients were assessed using Deseq2 (V1.30.0) in R. Significant differences were defined as Benjamin Hochberg false discovery rate (FDR) < .01 and a log2 fold change > 1. The GO gene enrichment analysis was conducted using ClusterProfiler (V4.0.5). Significant difference was defined as Benjamin‐Hochberg FDR < .05.

### 2.12 Immunohistochemistry

BC specimens that were formalin fixed and paraffin embedded were cut into 4‐μm sections for immunohistochemical staining. Hematoxylin and Eosin staining was used to section these tissues, which were then again sectioned after being indicated with morphologically representative specific tumour regions. Endogenous peroxidase was inhibited by 3% hydrogen peroxide solution after all sections were dewaxed with xylene and ethanol. 1% bovine serum albumin for 10 min at room temperature, followed by overnight incubation at 4°C with the following primary antibodies: monoclonal antibody against FCER2 (CD23; 1:400; 60208‐2‐Ig; Proteintech), anti‐CD21 (1:400; 24374‐1‐AP; Proteintech), anti‐CD20 (1:400; 60271‐1‐Ig; Proteintech), anti‐CD4 (1:400; 67786‐1‐Ig; Proteintech), anti‐CD8 (1:400; 66868‐1‐Ig; Proteintech) and anti‐BCL6 (1:400; 66340‐1‐Ig; Proteintech). The slides were then treated for 30 min at room temperature with secondary antibodies (horseradish peroxidase anti‐mouse, PR30012, Proteintech), and particular signals were exhibited with 3‐3′diaminobenzidine substrate (DAB), counterstained with hematoxylin, dried and sealed.

### 2.13 Mature TLS were quantified using H&E and CD23 IHC staining

TLS was measured by BC pathologists who did not have access to all of the samples’ pathological and genomic data. Based on morphology, TLS is assessed in H&E stained sections; mature TLS is taken into account when lymphocytes form rounded aggregates at the edge of breast tissue that has been infiltrated by the tumour. TLS is computed when a specific number of circular lymphocyte aggregates are seen at the breast tissue boundary of tumour invasion, together with a minimal amount of CD23 expression. Without CD23 expression, lymphoid aggregates were not counted.

### 2.14 Consistency study of CD23 to detect mature TLS

To investigate whether CD23 Immunohistochemistry (IHC) can serve as a marker for mature TLS, we randomly selected 70 cases of BC tissue specimens from patients who underwent curative surgery at our institution between January 2018 and January 2020. We also collected their clinical and pathological data, as well as survival information. The primary endpoint of the study was disease‐free survival (DFS), defined as the time interval from surgery to the first occurrence of invasive recurrence (local or distant), contralateral BC, or death due to any cause.

Following H&E and CD23 IHC staining, correlation test was performed to identify mature TLS in the entire tumour section using H&E and CD23. Furthermore, a BC pathologist who was unfamiliar with the relevant information of these slices counted the number of mature TLS on the entire tumour slice. At the same time, another pathologist also counted the mature TLS without knowing it. The consistency test was carried out by comparing the two results. The inter‐observer reliability and retest reliability were measured and evaluated using the intragroup coefficient, and the consistency of judgment was assessed using Cohen's Kappa.

### 2.15 Relevance of TAN and TIL‐B in BC

Marker gene expression of TAN and TIL‐B was analyzed in BC, and connection with other B cell markers was assessed. The Firehose Legacy dataset from TCGA consortium was used to produce gene expression data, which were then analyzed using the cBioportal Web application (https://www.cbioportal.org). There were 1108 samples from individuals with invasive BC that were all included. The cBioportal analysis tool was used to construct correlation charts that illustrate the relationship between the expression of a single gene and the expression of various genes. The analytic tool calculated the *p* values, Pearson correlations, and Spearman correlations.

### 2.16 Characterization of cell type infiltration based on scRNA‐seq

We used the online tool CIBERSORTx^16^ to create a reference feature matrix from our scRNA‐seq dataset and then estimated the proportion of cell types from independent BC cohorts (GSE7390, GSE58812) based on the constructed cell types in order to determine the proportion of the six major cell types we defined from bulk RNA‐seq data. There are 89 761 cells total in our reference data set of the six primary cell kinds, each of which is annotated as the primary cell type we previously discussed. When running CIBERSORTx on the RNA‐seq data set, the quartile normalization is turned off, the displacement parameter is set to 1000 times, and all other parameters are left at their default values in order to create the signature mix. Significant correlation is defined as having absolute correlation coefficient greater than .3 and FDR less than .05. The Euclidean distance of cell infiltration among all cell types in BC cohorts was evaluated and visualized using Heatmap.plus's unsupervised clustering function in R.

### 2.17 Identification of TLS_high and TLS_low tumours from bulk RNA‐seq

With the use of the bulk RNA‐seq data set, we inferred the TLS abundance using a technique akin to cell subtype deconvolution (GSE35640; GSE25055; IMvigor210). This research is based on the widely used 12‐CK labeling approach to find TLS characteristics in different cancers. Chemokine‐encoding genes CCL2, CCL3, CCL4, CCL5, CCL8, CCL18, CCL19, CCL21, CXCL9, CXCL10, CXCL11 and CXCL13 are part of the approach.^17^ The log‐transformed transcript copy count of the aforementioned characteristic genes is added to determine the abundance of TLS in tumours. Based on the calculation's findings, the aforementioned three BC cohorts are sorted into two groups using the median: TLS_ High and TLS_ Low. For visualization of the results of the calculation, use the survival function and ggplot function from the R package.

### 2.18 Statistics

The statistical analysis was performed using SPSS 16.0 and R software. Independent sample *t*‐tests and Mann Whitney *U*‐tests are used to assess statistical differences in the data, which are expressed as mean ± standard error of mean. The Kappa consistency test was used to determine the relationship between the two variables. When *p* < .05, statistical differences were considered significant.

## 3 RESULTS

### 3.1 Single‐cell expression profile and cell types of human BC

We conducted scRNA‐seq of primary tumour lesions and nearby normal tissues in 14 BC patients (14 women, 34−76 years) to investigate the microenvironment in BC (Figure 1). These 14 patients had different types of BC, including 3 with triple‐negative breast cancer (TNBC), 5 with Luminal A BC, 3 with HER‐2‐positive BC, and 3 with Luminal B BC, of which 10 lesions were infiltrative ductal carcinoma (IDC) and 4 lesions were ductal carcinoma in situ of the breast (DCIS) (Figure 1B). We collected a total of 124 587 cells from the single cell entire transcriptome after quality control assessment and the removal of duplicate cells,^15^ including 76 378 cells from primary BC lesions and 48 209 cells from nearby normal tissue (Figure 1A). Histological examination, IHC, and fluorescence in situ hybridization (FISH) were used to subtype the BC samples (Figure 1B).

All single cells were unsupervised grouped based on uniform flow UMAP analysis and the expression of their typical markers (Figure 1C). Seven major cell groups were then identified: neutrophils with high FCGR3B expression; T and NK cells with high CD3E expression; B cells with high CD79A expression; macrophages with high CD68 macrophage expression; epithelial cells with high EPCAM expression; fibroblasts with high COL3A1 expression; and endothelial cells with high PLVAP expression (Figure 1F, Figure S1B). Additionally, we observed that practically all cell populations were present in each individual lesion (Figure 1D) and that these cell populations were distributed unevenly among patients (Figure 1E). These findings demonstrate the extreme heterogeneity of human BC.

### 3.2 TIL‐B heterogeneity in BC

**FIGURE 2:**
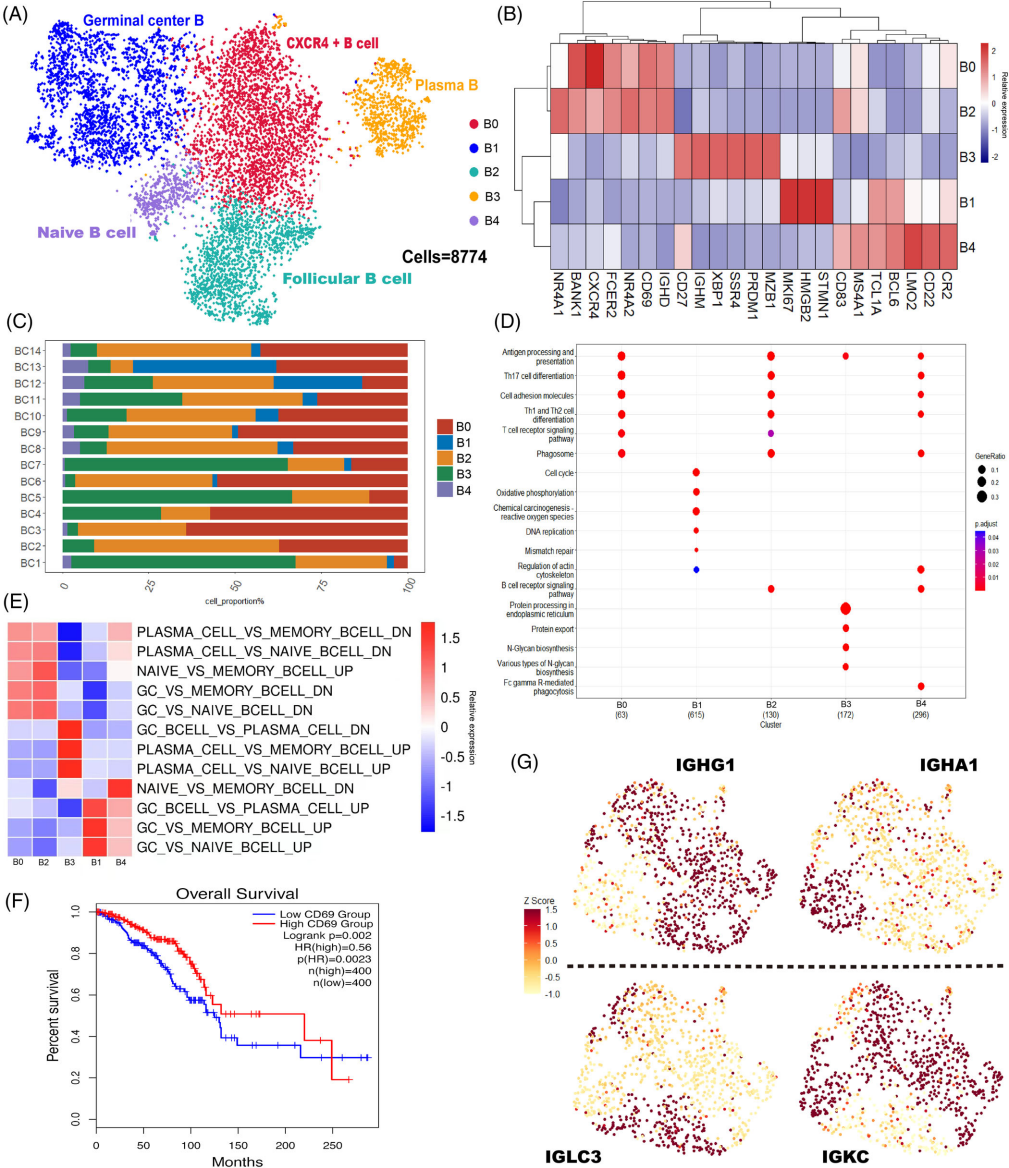
Tumour infiltrating B cells in breast cancer (BC). (A) In the current investigation, Umap projections of 8774 B cells from breast cancer patients revealed five significant B cell subgroups. (B) The B cell subcluster's corresponding distinctive genes are displayed on the heatmap. (C) The distribution of B cell clusters in the samples of 14 BC patients. (D) The GO enrichment pathways in B cell subsets are displayed in the dotplot. (E) Based on the heatmap‐displayed scRNA‐seq data of B cells, single sample GSEA (ssGSEA) was utilized to determine the cell identification of each cluster. (F) Survival curves were generated for BC patients categorized into high and low CD69 expression groups using the GEPIA (http://gepia2.cancer‐pku.cn/). (G) The expression of IGHG1, IGHA1, IGLC3 and IGKC in Cluster 3 (plasma) is shown using uniform manifold approximation and projection (UMAP) projection. GO, gene ontology; UMAP unified manifold approximation and projection; GSEA gene set enrichment analysis.

We found 8774 B cells in total, which we were able to group into five clusters (Figure 2A), with Cluster 0 being specifically characterized by relatively high levels of the chemokine receptor CXCR4; cluster 1 had relatively high expression of MKI67, a marker gene for germinal center (GC) B cells; cluster 2 had CD69, a protein linked to T‐cell activation, particularly abundantly expressed in follicular B cells; cluster 3 had relatively high expression of the plasma cell marker genes PRDM1, XBP1, MZB1 and SSR4; and cluster 4 had relatively high expression of the naive B‐cell marker gene TCL1A (Figure 2B). Significant disparities can be observed among B‐cell subpopulations across distinct BC molecular subtypes and individuals (Figure 2C and Figure S1A).

For the B‐cell subsets, we also conducted GO functional enrichment analysis (Figure 2D). CXCR4+ B cells (Cluster B0) were enriched in antigen presentation and processing and helper T‐cell differentiation (TH1, TH2 and TH17) signalling pathways; GC B cells (Cluster B1) were enriched in cell proliferation signalling pathways, such as DNA replication and mismatch repair, as well as oxidative phosphorylation signalling pathways. Follicular B cells (cluster B2) were enriched in T‐cell and B‐cell receptor signalling pathways; plasma cells (Cluster B3) were enriched in antigen presentation and processing signalling pathways in the endoplasmic reticulum; and naive B cells (Cluster B4) were enriched in major histocompatibility complex‐mediated antigen presentation and processing signalling pathways. The investigation of additional KEGG enrichment pathways was also done. We discovered that the B0 and B2 groups both exhibited a high degree of immune activation, whereas the B1 group was primarily controlled by DNA and RNA, the B3 group displayed a high degree of complement activation characteristics, and the B4 group was involved in the control of lymphocyte proliferation and immune response (Figure S1C–E). The transcription factor (TF) in each TIL‐B subgroup was also examined using SCENIC. TIL‐B once more demonstrated its incredibly paradoxical anti‐tumour and tumour‐promoting properties. The transcription factor ETS1, which is linked to a bad prognosis, was expressed by all subgroups. B1 and B4 also showed EZH2 boosting BC metastasis; however B0, B2 and B3 overexpressed ATF3 and FOXP1, which are related to a good prognosis (Figure S1F).

The DEGs of each B‐cell subpopulation were enriched using single sample gene set enrichment analysis (ssGSEA), and they were compared to the marker genes of several B‐cell subclasses in Hallmark (Figure 2E). According to these findings, Cluster B0 cells were a combination of plasma and GC B cells, and Cluster B1 cells were comparable to GC B cells. The cells in Cluster B2 were a mix of memory and naive B cells but skewed more toward memory B cells; plasma cells resembled cluster B3 cells, while naive B cells and GC B cells comprised cluster B4 cells.

Meanwhile, recent studies found that low CD69 expression was associated with poor survival of BC patients,^18^ and in our study, its correlation with overall survival (OS) was also verified using the TCGA database (Firehose Legacy) (Figure 2F). Importantly, we found that CXCR4+ B cells and follicular B cells relatively highly expressed CD69, while their functional enrichment showed mainly activation of immune‐related pathways, suggesting that both clusters may be associated with a better prognosis of BC.

The degree and composition of the immune response are key prognostic markers in BC, and there is evidence that the expression levels of distinct immunoglobulin light and heavy chains are related to the treatment result and prognosis of BC.^19^ We surprisingly observed mutual exclusion of immunoglobulin heavy‐ and light‐chain gene expression in plasma cells (Figure 2G) and investigated this phenomenon further (Figure S2). The heavy‐chain genes IGHG and IGHA as well as the light‐chain genes IGLC and IGKC displayed mutually exclusive differential expression in plasma cells and category‐converted B cells, respectively. This raises the possibility of localized sustained category switch recombination and the existence of underlying TLSs in BC.^20^ Additionally, we examined CD27+memory B cells in more detail and identified switched memory B cells (CD27+IGHD‐, 754/954 = 79.04%) and non‐switched memory B cells (CD27+IGHD+, 200/954 = 20.96%) (Figure S4A).^21^ This shows that TIL‐B shows obvious class conversion and affinity maturation in the tumour environment, which further shows the role of TIL‐B in anti‐tumour immunity and the possibility of the formation of TLSs.

### 3.3 Detailed analysis of TIL‐B reveals its maturation trajectory in BC lesions

**FIGURE 3:**
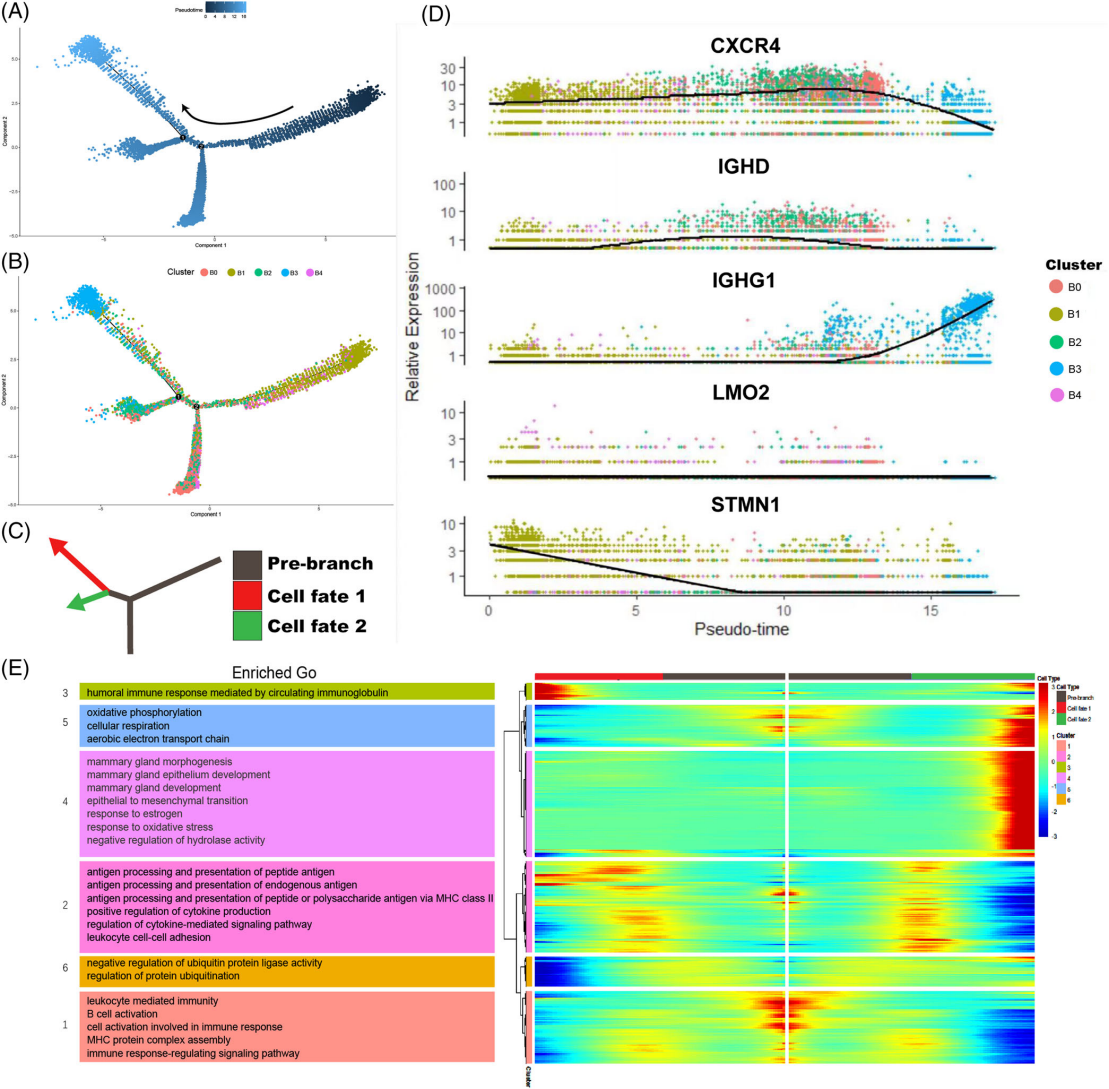
Analysis of the TIL‐B's developmental course in breast cancer (BC). (A and B) The pseudo temporal curve (A) and its development trajectory (B) of the TIL‐B subgroup are depicted in the pseudo temporal trajectory diagram of monocle 2. The development pathway of each subgroup of TIBs and the differentiation mechanism of TIBs as inferred by Monocle 2. (C) Two fictitious temporal paths of cell development are shown by red and green arrows. The track's starting before the branch point is indicated by the gray area. (D) Pseudo‐temporal change curve of genes with a high level of expression matching to each TIB subgroup. (E) DEGs reflecting the estimated pseudo time trajectory (in rows, *q*‐value  <  10^−10^) were hierarchically sorted into six sub clusters, and GO enrichment analysis was done on each sub cluster. DEGs, Differentially expressed genes; GO, gene ontology; TIL‐B, tumor‐infiltrating B cells.

All B‐cell trajectories were analyzed using Monocle 2 to determine how they could differentiate in the TME (Figure 3). The findings demonstrated that naive B cells (Cluster B4) and CXCR4+ B cells (Cluster B0) were in a transitional condition, follicular B cells (Cluster B2) and plasma cells (Cluster B3) were in the ultimate stage, and GC B cells (Cluster B1) were in the very early stage of B‐cell development. The results from earlier research showing that GC B cells can develop into plasma cells are compatible with this finding.^22^

Monocle discovered that GC B cells differentiated into two main branches, plasma cells and follicular B cells (Figure 3C), while GO enrichment analysis revealed (Figure 3E) that GC B cells were enriched in cell proliferation and regulation‐related pathways, whereas the two differentiated branches, plasma cells and follicular B cells, were enriched in immune response regulatory signalling pathways and BC regulation‐related pathways, respectively. At the same time, we also found that IGHG1 gradually increased at the later stage of the pseudo‐time trajectory (Figure 3D), which suggests that B cells underwent immunoglobulin conversion to develop into plasma cells.^23^ CXCR4 and STMN1,^24^, ^25^, ^26^ two genes that can boost immune function and encourage tumour spread, were simultaneously decreased gradually from an initial high expression to a final drop (Figure 3D), which also illustrates the contradictory antineoplastic and protumour capabilities of B cells.^11^

### 3.4 Correlation study of TIL‐B with mature TLS

**FIGURE 4:**
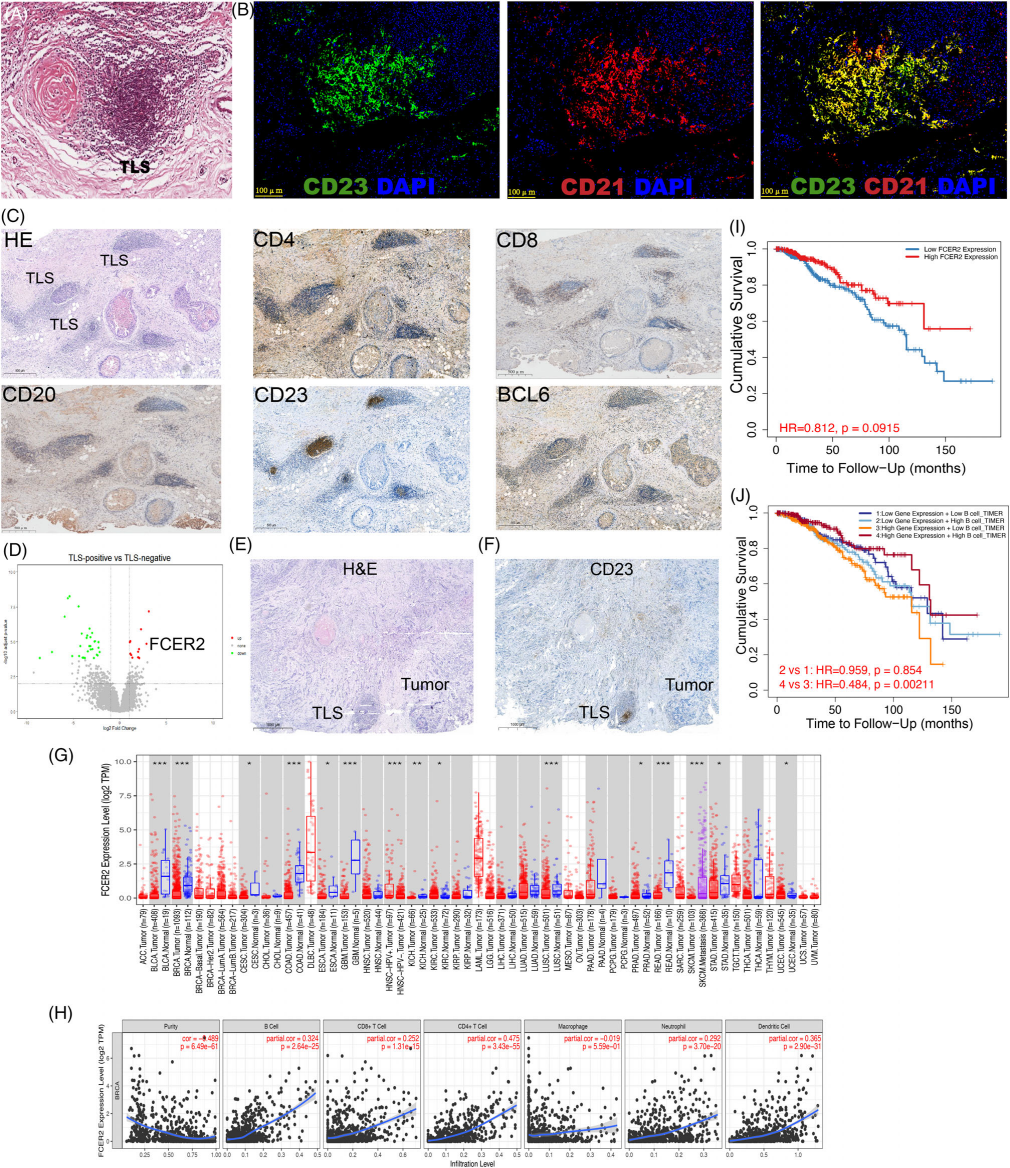
Correlation analysis between TIL‐B and mature tertiary lymphoid structures (TLS). (A) Among the 200 patients included in the BRCA dataset from the TCGA research consortium, a subset of 18 patients who exhibited TLS positivity were identified. H&E images of characteristic TLS in these patients. (B) Co‐immunofluorescence subimages depicting the co‐localization of CD23 and CD21 within CD23‐positive TLS were obtained from a collection of 70 BC samples that we compiled. (C) In 9 TLS‐positive patients of 70 BC patients, representative images of H&E staining and IHC staining of CD4, CD8, CD20, CD23 and BCL6 expression in TLS‐positive BC patients. (D) Analysis of DEGs in 200 TCGA BRCA cases categorized as TLS‐negative and TLS‐positive. Source data are available at: https://portal.gdc.cancer.gov. (E and F) H&E (E) and CD23 IHC (F) images of the typical TLS‐positive case in 70 BC patients. (G) Different tumour types exhibit FCER2 expression, according to TIMER2. *p < .05;**p < .01;***p < .001. (H) Utilizing the EPIC and TIDE algorithms within TIMER2, the relationship between FCER2 expression and tumour infiltrating immune cells in BC was computed. (I‐J) Kaplan–Meier Survival curve of FCER2(I), co‐acting with FCER2 and B cells(J) in BC by TIMER2. TIL‐B, tumor‐infiltrating B cells.

The connection between TIL‐B and TLS was our next area of investigation. Because of their significance in prognosis and treatment response, as well as their participation in the anti‐tumour immune response, TLSs are of interest.^27^ The presence of class‐switched B cells and GC B cells signifies that B cells may have undergone class‐switch recombination and somatic hypermutation. These evidences point to the possibility of the formation of TLSs.^20^ TLS is a heterotopic immune cell aggregation state that is characterized by the presence and interaction of different cell types. It is primarily made up of B cells, which are frequently surrounded by T cells and dendritic cells (DCs). To further examine this hypothesis, we used H&E‐stained tissue sections from the TCGA BRCA cohort to determine the presence of TLSs (Figure 4A). CD23 has recently been identified as a marker of follicular dendritic cells (FDC), and its expression on FDCs is not dependent on CD23 overexpression in tumours. As a result, immunofluorescence revealed that CD23 and the FDCs marker CD21 are co‐located (Figure 4B; Figure S5B). Additionally, numerous studies have demonstrated that mature TLSs also expresses CD23.^28^, ^29^ The IHC detection of continuous tissue sections allowed us to clearly see that TLS is primarily present near the BC, that CD20+B cells make up the majority of TLS, that they are surrounded by CD4+ T cells, CD8+ T cells and CD23+ FDCs, and that mature TLS has a significant concentration of BCL6+ GC B cells in the center. Unexpectedly, we also noticed that CD23 is strongly expressed in the GC of mature TLS (Figure 4C; Figure S5A). Additionally, differential gene analysis supported our findings that FCER2 (CD23) expression was significantly higher in BC with TLSs (Figure 4D; Table S1).

To investigate whether CD23 could be a marker for mature TLSs, two pathologists examined 70 BC slides from Yunnan Cancer Hospital with H&E and CD23 IHC staining (Figure 4E,F; Figure S5C,D). Among them, 21% of the H&E‐stained slides indicated uncertainty in distinguishing lymphoid aggregates from TLSs, but CD23 expression was not identified in lymphoid aggregates with immature TLS morphology. There was only modest agreement between the two approaches (intragroup coefficient .75, 95% CI .63‐.84; kappa .75, SE .089, Table S2), and the average number of mature TLSs per slide screened by H&E was much lower than that of CD23 (mean .66 vs. 1.2). The detection of mature TLSs using CD23 improved the interobserver agreement (intragroup coefficient .93, 95% CI .88–.96; kappa .86, SE .066, Table S2).

The TCGA dataset was then used to analyze the gene expression of FCER2 (CD23), which revealed elevated FCER2 mRNA expression in various cancers relative to normal tissues (Figure 4G), with BC having considerably greater FCER2 expression than the equivalent normal tissues. Tumour‐infiltrating immune cells that have invaded the tumour are crucial for tumour growth, recurrence, metastasis, and the effectiveness of immunotherapy.^30^ The EPIC and TIDE algorithms were used to evaluate the relationship between CD23 expression and major immune cell infiltration in BC. CD23 expression was found to be associated with the infiltration of B cells, CD8+ T cells, CD4+ cells, neutrophils, macrophages and dendritic cells (Figure 4H). We also discovered that only CD23 overexpression in BC has no prognostic value (Figure 4I), but CD23+B cells are associated with a higher rate of BC survival (Figure 4J).

### 3.5 The impact of TLS status on cellular interactions within the TME

To gain a deeper understanding of the TME, we delineated the proportion of fibroblast and immune cell populations. The research findings indicate substantial disparities in the proportions of fibroblast clusters and immune cell clusters between tumour and normal tissues (Figure S4B). Furthermore, variations in the distribution of different immune cell clusters and fibroblast clusters were observed among patients (Figure S4C). For instance, while normal tissue samples contained more neutrophils and fibroblasts, tumour samples contained more B cells, T cells, and macrophages. This indicates that BC cells have stronger chemotaxis to B cells, T cells and macrophages.

We first assessed the TLS status of 14 patients who had already undergone single‐cell tests to better understand the precise consequences of different states of TLS for BC patients. A total of nine patients were included in the follow‐up study due to the restricted number of pathological sections available from some of our patients. After H&E and IHC examination, three patients were confirmed to be TLS‐positive, while the other six patients were TLS‐negative.

**FIGURE 5:**
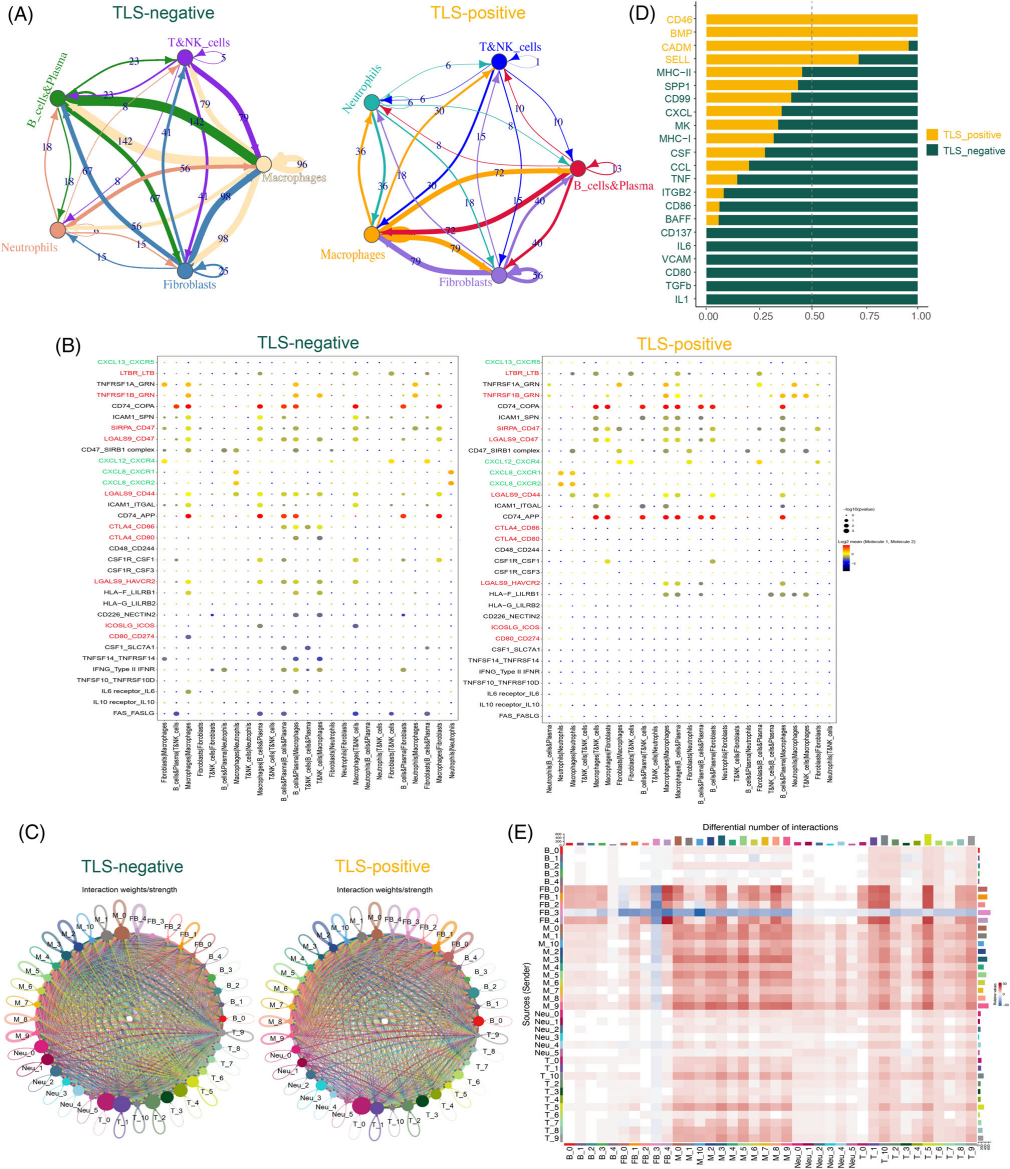
Cellular communication network between tumour microenvironment (TME). (A) Circos plot, utilizing CellphoneDB, is employed to elucidate the interaction between ligands and receptors in fibroblasts and diverse immune cell types. The analysis focuses on the tertiary lymphoid structures (TLS)‐negative (left) and TLS‐positive (right) cohorts of 9 breast cancer (BC) patients. (B) The Dotplot provides a visual representation of the intricate interplays among specific ligand‐receptor pairs within the TLS‐negative (left) and TLS‐positive (right) microenvironments, involving both fibroblasts and immune cells. This analysis was conducted using CellphoneDB. The red color denotes the receptor‐ligand pairs associated with immune checkpoints, while the green color signifies the receptor‐ligand pairs associated with chemokines. (C) Circular diagrams depicting the interaction weights between fibroblasts and immune subsets in the context of TLS‐negative (left) and TLS‐positive (right) conditions. (D) Bar graph of overall signal pathway intensity comparison: the yellow signal pathway is enriched in TLS‐positive, and the green signal pathway is enriched in TLS‐negative. (E) Heatmap of the differences in the number of interactions between TLS‐positive and TLS‐negative involving Fibroblasts and immune subsets. These diagrams were generated using CellChat.

In order to investigate the intricate cellular behaviors and intercellular communication within TLS‐positive and TLS‐negative groups, we utilized the expression profiles of receptors and ligands specific to each cell type to predict the potential interactions between distinct cell populations. Subsequently, we constructed putative intercellular communication networks for fibroblasts and immune cells in both groups (Figure 5A). Evidently, in the murine pancreatic milieu, the chemokine CXCL12 has demonstrated the capability to elicit site‐specific formation of TLS. A recent investigation presents compelling evidence derived from human BC, substantiating the role of tumour‐infiltrating CXCL13‐producing (CXCR5–) T follicular helper (TFH) cells, henceforth denoted as TFHX13 cells, in facilitating localized differentiation of memory B cells. TFHX13 cells possess the potential to initiate the formation of TLS, thereby engendering GC B cell responses at the tumour site.^31^ However, it is noteworthy that the TLS induced by CXCL13 markedly differs from the TLS induced by CXCL12, with the latter primarily consisting of minute aggregates of lymphocytes.^8^ Similarly, in the current study, TLS‐positive cells were discovered to express relatively high levels of CXCL12‐CXCR4 receptor ligand pairs, and small lymphoid aggregates along with FDC infiltration were observed in the corresponding immunohistochemical sections (Figure 4C). TLS‐positive cells also expressed relatively low levels of the FAS‐FASLG receptor‐ligand pairs, which are linked to immune tolerance. The immune checkpoint‐associated receptor‐ligand pairs CTLA4‐CD86, CTLA4‐CD80, ICOSL‐ICOS, LGALS9‐HAVCR2, CD80‐CD274 and LAGALS9‐CD44 were relatively more abundant in TLS‐negative cells than in TLS‐positive cells (Figure 5B). These findings point to a potential immunosuppressive microenvironment in TLS‐negative patients.

We systematically examined the differential interactions between fibroblast subpopulations and immune cell subpopulations at the ligand‐receptor level, signalling pathway level, and communication network level using CellChat in TLS‐positive and TLS‐negative groups. We discovered significant differences in cell interaction weight and number between TLS‐positive and TLS‐negative groups. For example, when compared to TLS positive, Cluster Fibro‐4 in TLS negative demonstrated strong interaction with various fibroblast subsets (Figure 5C,E). Cluster Fibro‐4 expressed high levels of MMP11 and was identified as myofibroblast (Table S3). According to recent research, this Fibroblast phenotype can increase the levels of PD‐1 and CTLA4 proteins in regulatory T lymphocytes (Tregs), which is related to cancer immunotherapy drug resistance.^32^ By comparing the differences in their signal pathways, we discovered that the co‐inhibition checkpoints (CD86, CD80, BAFF and CD137) and cytokines (transforming growth factor‐β [TGF‐β]) involved in the immunosuppressive process were up‐regulated in the TLS‐negative group,^33^ further demonstrating the existence of the immunosuppressive microenvironment in this group (Figure 5D).

### 3.6 scRNA‐seq reveals the plasticity of TAN under different states of TLS

**FIGURE 6:**
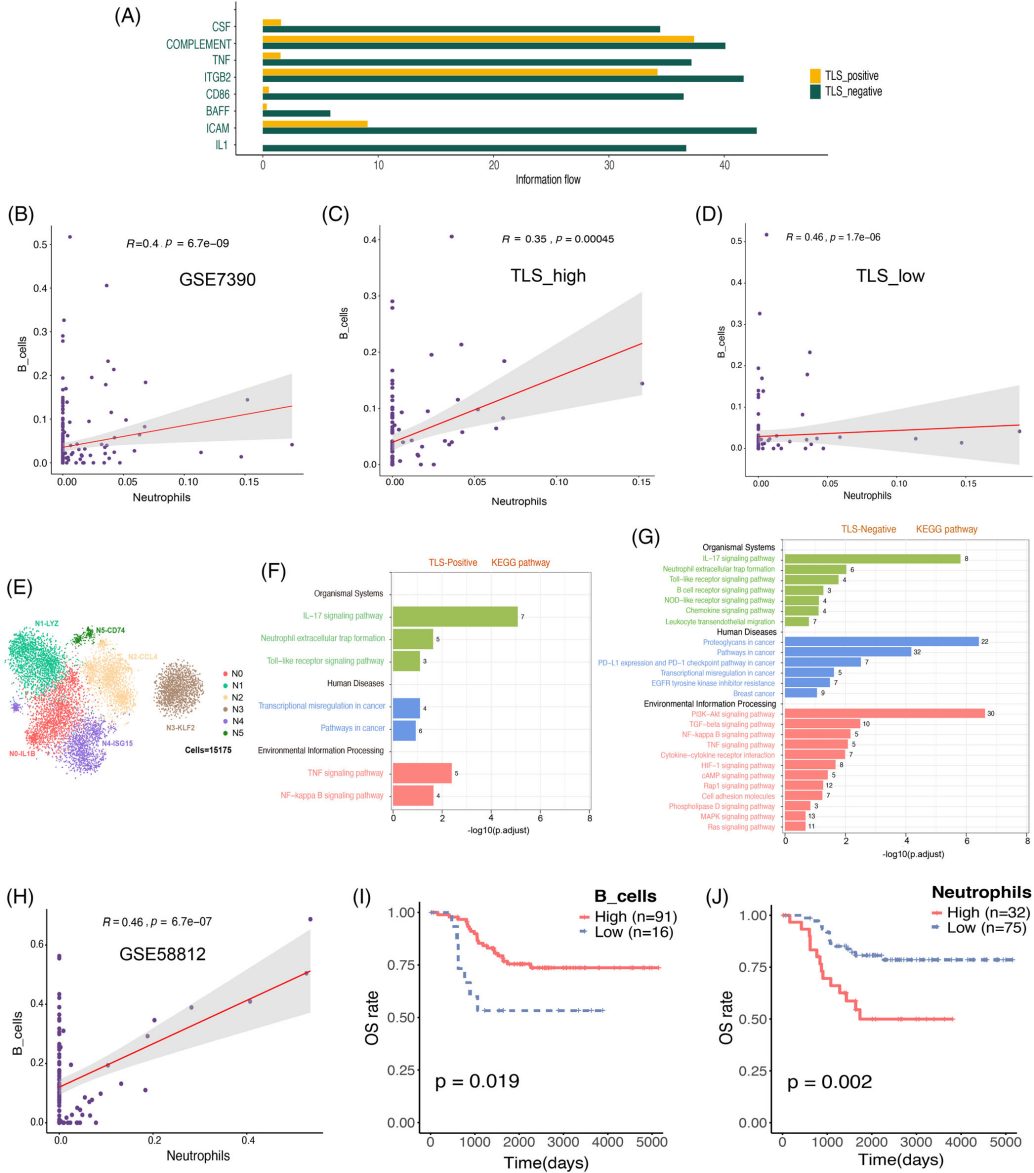
The flexibility of neutrophils in breast cancer (BC) was demonstrated by single cell sequencing. (A) The bar graph compares the overall intensity of signal pathways in 9 BC patients using data from CellChat. It reveals that the tertiary lymphoid structures (TLS)‐positive condition is characterized by a prominent yellow signal pathway, whereas the TLS‐negative condition is associated with a prominent green signal pathway. (B) The scatter figure depicts the relationship between tumour‐associated neutrophils (TAN) and TIL‐B infiltration in the BC cohort (GSE7390). The error band represents the 95% confidence interval. (C and D) The scatter chart depicts the association between TAN and TIL‐B infiltration as TLS high (C) and TLS low (D) in the BC cohort (GSE7390). The error band represents the 95% confidence interval. (E) Uniform manifold approximation and projection (UMAP) projections of 10 445 neutrophils from the BC patients that are now being researched, displaying six main subpopulations of neutrophils. (F and G) The neutrophil subgroup of 9 BC patients, comprising TLS‐Positive (F) and TLS‐Negative (G), is depicted through a bar chart illustrating the enriched outcomes of their primary KEGG pathways. The numerical values within the bar chart represent the quantity of genes encompassed within the relevant pathways. (H) The scatter plot depicts the relationship between the infiltration of TAN and TIL‐B in the triple‐negative BC (TNBC) queue (GSE58812). The error band represents the 95% confidence interval (CI). (I) The Kaplan–Meier curve revealed that in the TNBC cohort (GSE58812), patients with increased B cell infiltration had a higher OS. (J) The Kaplan–Meier curve revealed that patients with significant neutrophil infiltration had a worse chance of survival in the TNBC cohort (GSE58812). TIL‐B, tumor‐infiltrating B cells.

Recent studies demonstrate that TAN actively contributes to the recruitment of B cells to the TME, that TNFα is the primary cytokine mediating B cell chemotaxis to TAN, and that membrane B cell activating factor (BAFF) on TAN is a potential contact mechanism mediating B cell differentiation.^11^ We discovered an intriguing phenomenon in our study: TAN in the TLS‐negative group had relatively high levels of BAFF and TNF (Figure 6A). Because our scRNA‐seq dataset had a small sample size, we used the deconvolution algorithm CIBERSORTx to simulate the cell type‐specific gene expression profile and predict the abundance of each cell type in the large‐scale scRNA‐seq dataset. We trained CIBERSORTx on our own scRNA‐seq dataset to predict the robustness of cell type‐specific gene expression profiles from the gene expression omnibus data base dataset (Figure S3C,D). To better understand the relationship between TAN and TIL‐B in the BC microenvironment, we examined the paired Spearman correlation in the infiltration patterns of two cell types and their subsets in the BC cohort,^34^ dividing them into two groups: TLS‐low and TLS‐high (Figure 6B). When compared to TLS‐high, TAN and TIL‐B in the TLS‐low group had a significant positive correlation (Figure 6C,D).

For a long time, neutrophils were thought to be the main regulators of tumour progression due to their interaction with other infiltrating immune cells and subsequent regulation, but the identity of TAN remains a mystery. We identified TAN and classified it into six subtypes using the previously reported FCGR3B^11^ (Figure 6E). They include N0, which has a relatively high level of the pro‐inflammatory cytokine interleukin 1 beta protein (IL1B), N1, which has a relatively high expression of the lysozyme gene (LYZ), N2, which has a relatively high expression of the chemokine CCL4 and CCL3 genes, and N3, which has a relatively high expression of the inhibitory angioprotective factor KLF2 transcription factor, N4 with relatively high expression of ubiquitin‐like protein ISG15 gene and interferon inducible protein IFIT2 and IFLI3 gene and N5 with relatively high expression of class II major histocompatibility complex molecule‐related gene recombinant HLA Class II histocompatibility antigen (HLA‐DRA) (Figure 6E, Figure S3B). In addition, we also examined the distribution of TAN subgroups in different types of BC, and there was significant heterogeneity (Figure S3A).

Multiple factors in the TME drive functional plasticity in TAN.^12^ We also performed KEGG pathway enrichment analysis on TLS‐positive and TLS‐negative groups to better understand the effect of TLS expression level on TAN function. In comparison to the TLS‐positive group (Figure 6F), the TLS‐negative group has a higher level of inflammatory signalling pathways (Figure 6G), such as the PI3K‐AKt signalling pathway, the MAPK signalling pathway, the nucleotide‐binding oligomerization domain‐like receptor signalling pathway, and the NF‐kB signalling pathway. Recent research has linked local inflammation to immunosuppression, and an increase in inflammatory cells in the TME to ICB resistance.^35^ At the same time, the TLS‐negative group had a high level of PD‐L1 expression and the PD‐1 checkpoint path in cancer, as well as EGFR tyrosine kinase inhibitor resistance, TGF‐beta signalling pathway, and hypoxia‐inducible factor‐1 signalling pathway, all of which were linked to more invasive and treatment‐resistant cancer.^36^, ^37^, ^38^

Recent research has found that TAN is frequently found in the tumour niche of TNBC subtypes, whereas hormone receptor positive (HR+) BC with low invasiveness has less TAN, indicating that there is a link between the malignant potential of BC cells and the degree of neutrophil recruitment to tumours.^39^ The TNBC cohort was subjected to correlation and survival analysis in our study. TNBC, TAN and TIL‐B infiltration were discovered to be positively correlated. Simultaneously, the prognostic value of TAN and TIL‐B in HR+BC was insignificant (Figure S3E). TNBC patients with higher TIL‐B infiltration, on the other hand, had better OS and metastasis‐free survival (MFS) (Figure 6I, Figure S3F). Furthermore, higher TAN infiltration is linked to lower OS and MFS (Figure 6J, Figure S3F).

### 3.7 Different states of TLSs in BC exhibit metabolic heterogeneity and survival variations

**FIGURE 7:**
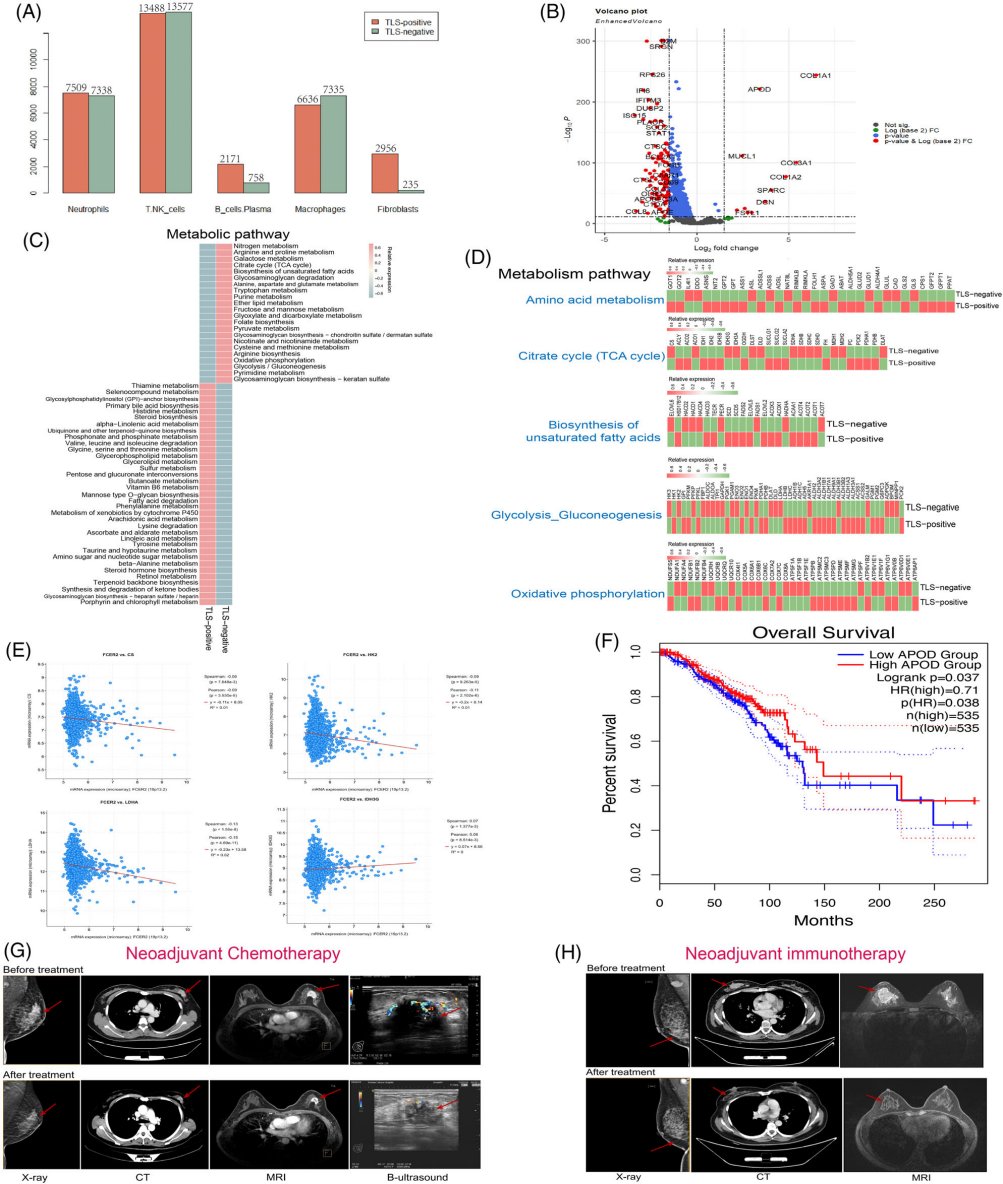
Different tertiary lymphoid structures (TLS) states in breast cancer (BC) display metabolic variability and survival variations. (A) A bar graph depicting the distribution of immune cells that have infiltrated tumours in TLS‐positive and TLS‐negative groups among 9 BC patients. (B) Genetic analysis of immune cell differences in TLS‐positive and TLS‐negative groups among 9 BC patients. (C) Heatmap of metabolic pathway scores in TLS‐positive and TLS‐negative groups among 9 BC patients. (D) Heatmap showing average metabolic gene expression in TLS‐positive and TLS‐negative groups among 9 BC patients. (E) To ascertain the expression correlation between CD23 and the biomarkers citrate synthase (CS), HK2, lactate dehydrogenase A (LDHA), and IDH3G in BC, the cBioportal tool (https://www.cbioportal.org) was utilized, employing the TCGA dataset (Firehose Legacy dataset; encompassing 1108 BC samples) for this purpose. (F) Kaplan–Meier survival curves depicting the disparity in BC outcomes between cohorts with high and low APOD expression, as analyzed through GEPIA. (G) Imaging before and after neoadjuvant chemotherapy of TLS‐positive patients with significant effectiveness. (H) Imaging before and after neoadjuvant immunotherapy of TLS‐positive patients with significant effectiveness.

We described the distribution of immune cells in different states of TLS (Figure 7A) and examined the composition of each immune subsets between the two groups (Figure S4E). We discovered that follicular B cells and CD8+T cells associated with good prognosis were enriched in TLS‐positive group.^40^ Meanwhile, we performed DEGs analysis of immune cells between the two groups (Figure 7B, Figure S4D). The results showed that the TLS‐positive group was relatively high in decorin, which inhibits angiogenesis and tumour proliferation,^41^ and APOD, which inhibits tumour growth,^42^ but it was also relatively high in COL1A1, which promotes tumour metastasis, highlighting the complexity of the TME once again.^42^

Then, we used the scMetabolism package to score the metabolic pathways and analyzed the gene expression of the two sets of immune cells at the single‐cell level. To our astonishment, TLS‐positive and TLS‐negative immune cells had entirely different metabolic pathways (Figure 7C). The tricarboxylic acid cycle (TCA cycle), glycolysis, and fatty acid production were all significantly increased in TLS‐negative tumours, and activation of these metabolic pathways is assumed to be closely related to carcinogenesis, progression and drug resistance.^43^ We concentrated on the TCA cycle, glycolysis, fatty acid biosynthesis, oxidative phosphorylation and amino acid metabolism in BC immune cells to further investigate the metabolic differences in various states of TLSs (Figure 7D), as metabolic reprogramming grants cancer cells the ability to survive and proliferate, a key factor in tumour development.^44^ The glycolysis rate‐limiting enzymes HK2, PKM, and LDHA,^44^ as well as the TCA cycle rate‐limiting enzymes CS, IDH3G, and IDH3A,^45^ were shown to have comparatively low expression in TLS‐positive cells. Additionally, we performed correlation analysis between these crucial metabolic rate‐limiting enzymes and CD23, the marker gene for TLSs. The results revealed that CS, HK2 and LDHA all displayed a strong negative connection with CD23, while IDH3G displayed a modest positive correlation with CD23 (Figure 7E). These data further show that multiple metabolic states exist in various TLS states.

Next, we conducted a survival analysis of APOD, a gene that is expressed differently in TLS‐positive and TLS‐negative patients, to examine the differences in OS between the various states of TLSs (Figure 7F). The results revealed that patients with high APOD expression, also known as TLS‐positive patients, had a relatively longer OS.

Meanwhile, TLS was discovered in the tumour tissues of two BC patients who had significant effects on neoadjuvant immunotherapy and neoadjuvant chemotherapy. The first TLS‐positive patient (Figure 7G) with Luminal B BC underwent neoadjuvant chemotherapy with a regimen of four cycles of EC (epirubicin + cyclophosphamide) combined with two cycles of T (albumin paclitaxel), and this patient achieved clinical remission after six cycles of treatment with good outcomes. Another TLS‐positive patient (Figure 7H) with TNBC achieved a considerable response after neoadjuvant immunotherapy with six cycles of carrelicizumab. According to these findings, TLS‐positive BC patients, even those with a molecular type of BC that is resistant to standard treatment, may respond better to chemotherapy and immunotherapy.

Furthermore, we discovered that one of the 70 patients with IHC received neoadjuvant chemotherapy, while the other received neoadjuvant immunotherapy. We collected clinicopathological information and image data from the two patients with negative‐TLS after reconfirming the TLS status in the pathological section. After carefully comparing the imaging data of the two patients before and after treatment, we obtained surprising results. The neoadjuvant chemotherapy given to the TLS‐negative patients in this study was not ideal. The patients showed stable condition (SD) after three cycles of albuminpaclitaxel + epirubicin + cyclophosphamide treatment. The neoadjuvant immunotherapy effect was not evident in another patient who tested negative for TLS. The patient was in SD after four cycles of Karelizumab treatment (Figure S7E, Table S5).

To validate our findings, we divided them into two groups based on 12 gene characteristics from three independent cohorts^46^, ^47^, ^48^ (GSE35640; GSE25055; IMvigor210): CD23+TLS_ Low and CD23+TLS_ High, and compared their response to neoadjuvant chemotherapy and immunotherapy (Figure S7A–C). In the neoadjuvant chemotherapy BC cohort, we discovered that CD23+ TLS _high patients demonstrated obvious chemosensitivity (*p* < .01, Figure S7B). At the same time, CD23+TLS high patients in the immunotherapy melanoma cohort showed significant ICB sensitivity (*p* < .01, Figure S7A). Furthermore, although there was no significant difference in immunotherapy response between the high and low groups in the IMvigor210 cohort^48^ (Figure S7C), the CD23+TLS_high group had a better clinical prognosis (Figure S7D).

### 3.8 The prognostic value of TLS and TLS‐specific markers in BC

**FIGURE 8:**
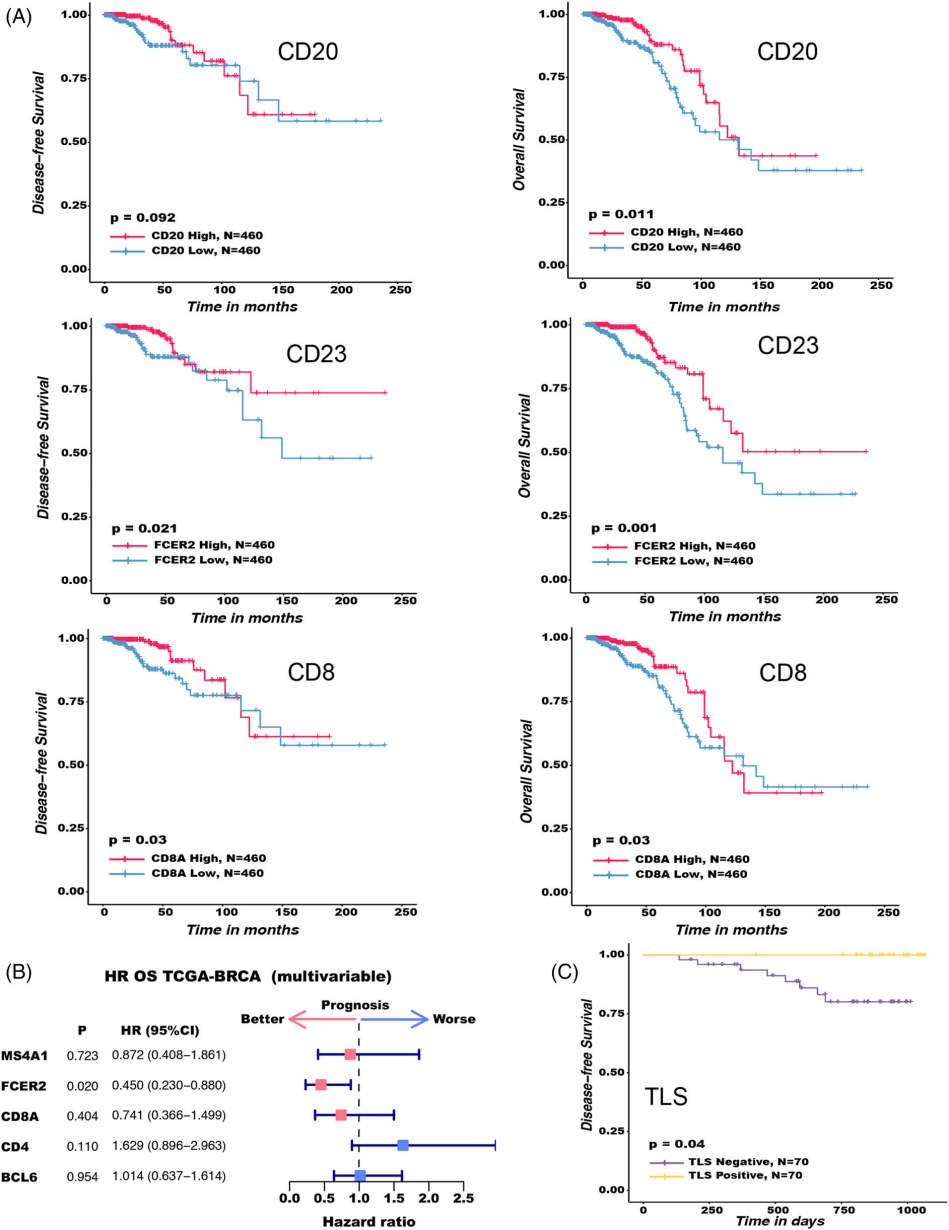
The potential significance of tertiary lymphoid structures (TLS) and TLS‐specific markers for breast cancer (BC) prognosis. After matching with Fitness score matching (PSM), survival analysis and prognostic factor analysis were performed. For survival analysis, the Kaplan–Meier method and the log‐rank test were employed. For prognostic factors, COX proportional hazard regression model was used. The two‐sided log rank test was used to determine the *p* values. (A) Kaplan–Meier survival curves for the disease‐free survival (DFS) (left) and OS (right) of 920 TCGA BRCA patients based on single gene expression (CD20, CD23 and CD8). (B) The impact of CD20, CD23, CD8, CD4 and BCL6 on 920 TCGA BRCA patients’ prognosis. The forest map shows HRs (center pink and blue squares) and 95% confidence interval (horizontal ranges), and PSM matching has been made for factors such as molecular typing, lymph node status, tumour size, diagnosis age and histological grading of BC. (C) Kaplan–Meier survival curves for the DFS of 70 BC patients based on TLS expression.

The prognostic significance of TLS‐specific markers (CD20, CD23, CD8, CD4 and BCL6) in TCGA BRCA patients was then assessed (Table S4). We discovered that CD4 and BCL6 expression levels did not significantly affect the OS and DFS of TCGA BRCA patients (Figure S6A), whereas CD20, CD23 and CD8 expression levels were positively correlated with OS and DFS (Figure 8A). More significantly, when CD23, a TLS‐specific marker, was used in univariate and multivariate cox risk regression analysis of TCGA BRCA patients, it outperformed the more widely used B cell marker CD20 in terms of risk ratio (HR) (Figure 8B, Figure S6B), suggesting that it may have superior prognostic value. We also performed a survival analysis on 70 BC patients who had undergone the TLS test at the same time. The findings indicated that TLS was a potentially beneficial factor for the prognosis of BC because it was significantly related to the improvement of DFS (Figure 8C).

## 4 DISCUSSION

TLS is a collection of heterotopic lymphocytes primarily created by B cell enrichment. Its existence could be linked to improved survival and treatment response, which could be linked to the fact that TLS promotes an effective immunological milieu.^20^ Based on scRNA‐seq, we demonstrated in this study that distinct cells in TME will display glaring intercellular communication and metabolic heterogeneity under diverse TLS conditions and have significant survival variations. Better chemotherapeutic efficacy or immunological treatment response are connected to mature TLS. In addition, utilizing CD23 as an immunohistochemical marker of mature TLS, TLS was discovered in 13% of BC patients and was not associated with clinicopathological features and molecular type.

The significance of B cells in cancer has drawn increasing attention. There is evidence that B cells infiltrate many different tumour types, including those of the lung, colon, ovary and liver.^49^ However, there is a variety of contradictory evidence about the function of B lymphocytes in solid tumours, indicating that they can either promote tumour progression or inhibit tumour growth.^50^, ^51^ Particularly, there has been substantial debate concerning the general functional significance of TIL‐B cells in human BC,^52^ which may be related to the diversity of B‐cell subtypes and BC patient populations. At the same time, as a major constituent of the immunological microenvironment, TAN exhibits functional flexibility influenced by a variety of TME variables. Its functional variations and molecular mechanisms under various TLS conditions are not completely understood.

To identify the intratumour and immune subtype‐specific characteristics of BC, we evaluated the immune cell population that infiltrated the tumour using single‐cell transcriptome sequencing. First, we detected seven major cell categories in a total of 124,587 cells, including neutrophils, T&NK cells, B cells, macrophages, epithelial cells, fibroblasts and endothelial cells. The majority of these cells are consistent with other investigations.^53^ TIL‐B and TANs were the subject of thorough single‐cell research since, in contrast to T cells, their phenotypic traits, functional impacts, and interactions have not been widely studied. TIL‐B was classified into five clusters and TANs into six clusters, and their potential biological functions were explored. We also discovered uncommon cell types, including follicular B cells. Follicular B cells in BC are the subset of B cells most related to immunotherapeutic effectiveness, according to research by Zhang et al.^40^ They also improve the prognostic role of T cells, which are typically correlated with a positive prognosis.

TLSs are ectopic lymphocyte aggregates primarily produced by B‐cell enrichment. B cells and TLSs may be associated with better survival and therapeutic responses, which may be connected to the fact that TLSs help to maintain an efficient immunological milieu.^54^ The possible prognostic relevance of TLSs and the specific molecular mechanisms are as yet unknown. The markers used to define and characterize TLSs vary by study, which complicates subsequent comparative investigations of TLSs. Additionally, it is challenging to translate findings from the tumour immune microenvironment study into clinical practice since the analytical techniques used in most studies are typically not appropriate for regular diagnosis. For instance, 12‐CK scores^55^ and 9‐gene signatures,^10^ which were discovered using transcriptome analysis, have been confirmed for use in the identification of TLSs, although the aforementioned techniques are challenging to put into practice. Moreover, the usage of H&E stained slides has a low repeatability and is quite individualized. Although simultaneous immunohistochemical labeling of TLSs trademark immune cell subpopulations is also possible, it is exceedingly labor intensive and time‐consuming to achieve. Therefore, to take advantage of TLS's diagnostic and possible predictive usefulness in various illness scenarios, we need to develop a simple and standardized way to measure TLS.

By analyzing B cells using scRNA‐seq, we were able to identify GC B cells and antibody‐secreting B cells. Antibody‐secreting B cells also underwent class transformation, demonstrating the presence of a persistent B‐cell response in BC and the development of TLSs. We assumed that CD23 is a potential marker of TLS because it is a marker of FDC and is also expressed in mature TLSs. This assumption was confirmed by IHC staining of subsequent tissue sections and DEGs analysis. The sections stained with CD23 IHC analysis demonstrated that mature TLS GC had robust CD23 expression, and that this expression was unrelated to whether CD23 was expressed within the tumour itself. Lymphoid structures expressing CD23 seemed to be mature TLSs with GC based on co‐immunofluorescence and IHC characteristic of immune cells. We examined tumour samples from 70 BC patients at Yunnan Cancer Hospital utilizing CD23 expression in lymphoid tissues as a marker and discovered that 13% of them had TLSs, with a high level of interobserver agreement. Recent research has demonstrated that the maturity of TLSs, particularly the existence of growth centers, can produce memory cells and high affinity, long‐lived plasma cells, which are crucial for patient prognosis.^56^ Only mature TLSs with GC morphology showed CD23 expression in the current investigation. In light of this, CD23 might be a useful marker for clinically significant TLSs. However, due to limitations in the research data, we were unable to conduct further analysis of the B cell receptor (BCR) to provide more direct evidence. This is also one of the limitations of this study. In future research, we will undertake a more comprehensive analysis of the BCR to acquire additional information.

Because TLS is an independent factor affecting survival, we further analyzed the cellular communication and metabolic difference of fibroblasts and immune cells in TLS‐positive and TLS‐negative groups to explore the impact of different TLS states on TILs and fibroblasts. We found that TLS‐positive group showed a better prognosis possibility whether from the analysis of cellular communication network or metabolic pathway. Cytokines involved in immunosuppression (TGF‐β) and co‐inhibition checkpoints (CD86, CD80, BAFF, CTLA4 and CD137) were up‐regulated in the TLS‐negative group, indicating that there was an immunosuppressive microenvironment in the TLS‐negative group. Tumour cells evade immune recognition by up‐regulating the expression of immune checkpoints on immunosuppressive cells.

It is interesting to note that TIL‐B in TLSs is linked to improved survival in pancreatic cancer patients but that this favorable prognostic effect is lost when these TIL‐B are randomly distributed throughout the tumour.^57^ This suggests that TLSs have a better prognostic value than TIL‐B cells, which is better demonstrated from a clinical perspective in our study, where we found that patients with Luminal B BC, who may not be sensitive to chemotherapeutic agents, were able to achieve better chemotherapeutic outcomes in the presence of mature TLSs, and similarly, patients with TNBC, who are less sensitive to ICB, were able to achieve better immunotherapeutic outcomes in the presence of mature TLSs. In our clinical practice, we verified the link between TLSs and treatment response, demonstrating that TLS‐positive patients are more likely to experience better treatment outcomes whether they receive chemotherapy or immunotherapy. TIL‐B has been shown in studies to secrete antibodies and cytokines, participate in antigen presentation, and regulate T cell function and then play a role in tumour promotion or anti‐tumour, depending on whether TIL‐B is in immature TLS or mTLS. TIL‐B may release into TME or express a series of molecules on its membrane in immature TLS, weakening the effective anti‐tumour immune response. TIL‐B, on the other hand, can present tumour‐derived antigen to CD8+T cells in mTLS, promoting anti‐tumour immunity. Although the precise mechanism of TLS anti‐tumour activity remains unknown, it is now clear that plasma cells are generated in TLS GC, which can produce anti‐tumour antibodies and form antigen‐antibody complexes with tumour‐related antigens, which are then internalized by DC and effectively present antigens to T cells. This amplification mechanism can more effectively activate CD8+T cells in TME, especially when the immune checkpoint is blocked.

TAN exhibits functional flexibility as a result of numerous variables present in the TME.^12^ In our research, we discovered an intriguing occurrence in which the state of TLS influences the function of TAN. TAN has a higher level of inflammatory reaction and treatment resistance in the TLS‐negative group, which is associated with a bad prognosis.^35^, ^36^ Current research has indicated that TAN is common in TNBC, but hormone receptor positive (HR+) BC with low invasiveness contains less TANs. We believe that TAN infiltration may be a predictor of poor prognosis and ICB responsiveness in TNBC.

However, some of our findings have not been published yet, as further functional validation is still required. Additionally, our approach has certain limitations and potential confounding factors, such as the inability to provide direct information regarding immune cell abundance or composition in the TME, as well as potential inter‐individual variability in the expression of immune cell markers. Despite these limitations, our study may still serve as a valuable resource for future research and, as a proof‐of‐concept study, has opened doors for further treatment options for BC patients. Furthermore, we conducted a more detailed prognostic analysis of TLS and its specific markers. The research findings demonstrate a significant association between CD20, CD23, CD8, TLS and favorable prognosis in BC patients. To further confirm the prognostic value of TLS, additional clinical correlations should be conducted using larger cohorts in the future.

As a result of our more in‐depth research into the tumour immune microenvironment and our analysis of the heterogeneity of the TIL‐B and TAN subpopulations, we are now better able to understand the general functional roles that these cells play in the development and treatment of cancer. However, more research is required to understand the regulatory and intricate molecular processes that TIL‐B and TANs go through. We also assessed the functional role of B cells in TLSs and the potential prognostic role of TLSs in tumours, demonstrated the presence of mature TLSs and provided a straightforward marker to identify TLSs. Future validation experiments will be conducted to achieve even more useful results.

## 5 CONCLUSION

In conclusion, our study highlights the heterogeneity of B cells in BC, explains how B cells and TLSs contribute significantly to anti‐tumour immunity at both the single cell and clinical levels and offers a straightforward marker for TLSs called CD23. These results will offer more pertinent information on the applicability and effectiveness of tumour immunotherapy.

## CONFLICT OF INTEREST STATEMENT

All authors have completed the ICMJE uniform disclosure form. The authors have no conflict of interest to declare.

## Supporting information

(A) The tSNE plot illustrates the expression patterns of TIL‐B across distinct molecular subtypes within a cohort comprising 14 BC patients.
(B) Utilizing multi‐marker genes, we employed Dotplot visualization to illustrate the diverse cellular types in 14 BC patients.
(C) Pathway enrichment analysis based on the KEGG was conducted to investigate the TIL‐B subsets in 14 BC patients.
(D and E) Diagram of chord illustrating the specific GO functional enrichment of each TIL‐B subgroup in 14 BC patients.
(F) TIL‐B SENIC study in 14 BC patients. tSNE plot displaying the regulatory intensity of distinct transcription factors (TFs) in TIL‐B (left) and heat map displaying the regulatory intensity of TFs in TIL‐B (right).

**Figure S2 Heavy and light chain expression in B cells as a whole**. The UMAP projection reveals IGHG1, IGHG2, IGHG3, IGHG4, IGHA1, IGHA2, IGHE, IGHD, IGHM, IGLC2, IGLC3, and IGKC expression in TIL‐B within 14 BC patients.

**Figure S3 Functional status of TAN subpopulations in BC**.
The tSNE plot illustrates the expression patterns of TAN across distinct molecular subtypes within 14 BC patients.
(A) The TAN sub‐clusters' highly expressed genes in 14 BC patients are shown on the heatmap.
(B) UMAP display of our scRNA‐seq data from 14 BC tissues (upper) and method for evaluating single‐cell deconvolution performance (bottom).
(C) Concordance of cell type proportions of six major cell types as determined by CIBERSORTx deconvolution.
(D) The Kaplan–Meier curve revealed that the degree of B cell and neutrophil infiltration was not significantly linked with OS and metastasis‐free survival (RFS)in the BC cohort (GSE7390).
(E) The Kaplan–Meier curve revealed that in the TNBC cohort (GSE58812), patients with higher B cell infiltration had a better RFS (upper), while patients with higher neutrophil infiltration had a worse RFS (bottom).

**Figure S4 Detailed characterization of Immune cells in BC**.
(A) The tSNE plot depicts the relative proportion of non‐switched memory B cells (left) and switched memory B cells (right) in 14 BC patients.
(B) The compositions between normal and tumour tissues for immune cells in 14 BC patients.
(C) The proportiions of immune cells between normal (left) and tumour (right) tissues in 14 BC patients.
(D) DEGs analysis for each immune subsets in TLS‐positive and TLS‐negative groups of 9 BC patients.
(E) The composition difference between TLS‐positive and TLS‐negative groups of 9 BC patients for each immune subsets.

**Figure S5 Expression of TLSs and CD23 in BC**.
(A) Representative overview and details of H&E staining and IHC staining of CD4, CD8, CD20, CD23 and BCL6 expression in TLS‐positive BC patients. The images in the black rectangle box correspond to the images in Figure 5C, and the images in the red rectangle box correspond to the TLS details on their right.
(B) Overview of CD23 and CD21 co‐immunofluorescence analysis in CD23‐positive TLS. The position of the orange rectangle corresponds to the image in Figure 5B.
(C and D) Overview of H&E (C) and CD23 IHC (D) images of the typical TLS‐positive case. The images in the black rectangle box correspond to the images in Figure 5E‐5F, and the images in the red rectangle box correspond to the TLS details on their right.

**Figure S6 Survival analysis of BRCA patients in the TCGA dataset**.
(A) Kaplan–Meier survival curves for the DFS (left panels) and OS (right panels) of 920 TCGA BRCA patients based on single gene expression (CD4 and BCL6).
(B) The forest map shows HRs (center pink and blue squares and circles) and 95% confidence interval (horizontal ranges), and PSM matching has been made for factors such as molecular typing, lymph node status, tumour size, diagnosis age and histological grading of BC.

**Figure S7 The presence of mature TLS is associated with better patient survival and treatment response**.
(A–C) In three separate immunotherapy cohorts, GSE35640 (melanoma), GSE25055 (BC) and IMvigor210 (urothelial carcinoma), the association between TLS and treatment response is depicted by the circle diagram.
(D) The presence of mTLS is related to better OS in IMvigor210, according to the Kaplan–Meier curve.
(E) Imaging examinations demonstrate the therapeutic efficacy in TLS‐negative patients pre‐ and post‐administration of neoadjuvant chemotherapy (left) and neoadjuvant immunotherapy (right).

**Table S1 Differential gene analysis between TLS‐positive and TLS‐negative**.

**Table S2 Correlation test and consistency test of mature TLS expression in BC**.

**Table S3 Gene expression of each immune subsets**.

**Table S4 Analysis of prognostic factors in BC**.

**Table S5 Clinical and pathological features of patients with and without TLS**.

## Data Availability

The dataset supporting the conclusions of this article is available in the Figshare repository, https://doi.org/10.6084/m9.figshare.21737615.

## References

[1] Yamazaki CM , Yamaguchi A , Anami Y , et al. Antibody‐drug conjugates with dual payloads for combating breast tumor heterogeneity and drug resistance. Nat Commun. 2021;12(1):3528. 34112795 10.1038/s41467-021-23793-7 PMC8192907

[2] Gonzalez Castro LN , Tirosh I , Suvà ML . Decoding cancer biology one cell at a time. Cancer Discov. 2021;11(4):960‐970. 33811126 10.1158/2159-8290.CD-20-1376 PMC8030694

[3] Hinshaw DC , Shevde LA . The tumor microenvironment innately modulates cancer progression. Cancer Res. 2019;79(18):4557‐4566. 31350295 10.1158/0008-5472.CAN-18-3962 PMC6744958

[4] Thommen DS , Schumacher TN . T cell dysfunction in cancer. Cancer Cell. 2018;33(4):547‐562. 29634943 10.1016/j.ccell.2018.03.012 PMC7116508

[5] Lu Y , Zhao Q , Liao J‐Y , et al. Complement signals determine opposite effects of B cells in chemotherapy‐induced immunity. Cell. 2020;180(6):1081‐1097. 32142650 10.1016/j.cell.2020.02.015

[6] Bruno TC , Ebner PJ , Moore BL , et al. Antigen‐presenting intratumoral B cells affect CD4 TIL phenotypes in non‐small cell lung cancer patients. Cancer Immunol Res. 2017;5(10):898‐907. 28848053 10.1158/2326-6066.CIR-17-0075 PMC5788174

[7] Shen M , Wang J , Ren X . New insights into tumor‐infiltrating B lymphocytes in breast cancer: clinical impacts and regulatory mechanisms. Front Immunol. 2018;9:470. 29568299 10.3389/fimmu.2018.00470 PMC5852074

[8] Schumacher TN , Thommen DS . Tertiary lymphoid structures in cancer. Science. 2022;375(6576):eabf9419. 34990248 10.1126/science.abf9419

[9] Franzoi MA , Romano E , Piccart M . Immunotherapy for early breast cancer: too soon, too superficial, or just right? Ann Oncol. 2021;32(3):323‐336. 33307202 10.1016/j.annonc.2020.11.022

[10] Wang B , Liu J , Han Y , Deng Y , Li J , Jiang Y . The presence of tertiary lymphoid structures provides new insight into the clinicopathological features and prognosis of patients with breast cancer. Front Immunol. 2022;13:868155. 35664009 10.3389/fimmu.2022.868155 PMC9161084

[11] Shaul ME , Zlotnik A , Tidhar E , et al. Tumor‐associated neutrophils drive B‐cell recruitment and their differentiation to plasma cells. Cancer Immunol Res. 2021;9(7):811‐824. 33906865 10.1158/2326-6066.CIR-20-0839

[12] Shaul ME , Fridlender ZG . Neutrophils as active regulators of the immune system in the tumor microenvironment. J Leukoc Biol. 2017;102(2):343‐349. 28264904 10.1189/jlb.5MR1216-508R

[13] Swierczak A , Mouchemore KA , Hamilton JA , Anderson RL . Neutrophils: important contributors to tumor progression and metastasis. Cancer Metastasis Rev. 2015;34(4):735‐751. 26361774 10.1007/s10555-015-9594-9

[14] Stoeckius M , Hafemeister C , Stephenson W , et al. Simultaneous epitope and transcriptome measurement in single cells. Nat Methods. 2017;14(9):865‐868. 28759029 10.1038/nmeth.4380 PMC5669064

[15] McGinnis CS , Murrow LM , Gartner ZJ . DoubletFinder: doublet detection in single‐cell RNA sequencing data using artificial nearest neighbors. Cell Syst. 2019;8(4):329‐337. 30954475 10.1016/j.cels.2019.03.003 PMC6853612

[16] Newman AM , Steen CB , Liu CL , et al. Determining cell type abundance and expression from bulk tissues with digital cytometry. Nat Biotechnol. 2019;37(7):773‐782. 31061481 10.1038/s41587-019-0114-2 PMC6610714

[17] Coppola D , Nebozhyn M , Khalil F , et al. Unique ectopic lymph node‐like structures present in human primary colorectal carcinoma are identified by immune gene array profiling. Am J Pathol. 2011;179(1):37‐45. 21703392 10.1016/j.ajpath.2011.03.007 PMC3123872

[18] Maeda T , Hiraki M , Jin C , et al. MUC1‐C induces PD‐L1 and immune evasion in triple‐negative breast cancer. Cancer Res. 2018;78(1):205‐215. 29263152 10.1158/0008-5472.CAN-17-1636 PMC5754244

[19] Larsson C , Ehinger A , Winslow S , et al. Prognostic implications of the expression levels of different immunoglobulin heavy chain‐encoding RNAs in early breast cancer. NPJ Breast Cancer. 2020;6:28. 32656317 10.1038/s41523-020-0170-2 PMC7338507

[20] Dieu‐Nosjean M‐C , Goc J , Giraldo NA , Sautès‐Fridman C , Fridman WH . Tertiary lymphoid structures in cancer and beyond. Trends Immunol. 2014;35(11):571‐580. 25443495 10.1016/j.it.2014.09.006

[21] Janjic BM , Kulkarni A , Ferris RL , Vujanovic L , Vujanovic NL . Human B cells mediate innate anti‐cancer cytotoxicity through concurrent engagement of multiple TNF superfamily ligands. Front Immunol. 2022;13:837842. 35392082 10.3389/fimmu.2022.837842 PMC8983021

[22] Ise W , Fujii K , Shiroguchi K , et al. T follicular helper cell‐germinal center B cell interaction strength regulates entry into plasma cell or recycling germinal center cell fate. Immunity. 2018;48(4):702‐715. 29669250 10.1016/j.immuni.2018.03.027

[23] Moore EM , Maestas DR , Cherry CC , et al. Biomaterials direct functional B cell response in a material‐specific manner. Sci Adv. 2021;7(49):eabj5830. 34851674 10.1126/sciadv.abj5830 PMC8635437

[24] Liu J , Li J , Wang K , et al. Aberrantly high activation of a FoxM1‐STMN1 axis contributes to progression and tumorigenesis in FoxM1‐driven cancers. Signal Transduct Target Ther. 2021;6(1):42. 33526768 10.1038/s41392-020-00396-0 PMC7851151

[25] Zhang R , Gao X , Zuo J , et al. STMN1 upregulation mediates hepatocellular carcinoma and hepatic stellate cell crosstalk to aggravate cancer by triggering the MET pathway. Cancer Sci. 2020;111(2):406‐417. 31785057 10.1111/cas.14262 PMC7004522

[26] Zhang ED , Li C , Fang Y , et al. STMN1 as a novel prognostic biomarker in HCC correlating with immune infiltrates and methylation. World J Surg Oncol. 2022;20(1):301. 36127700 10.1186/s12957-022-02768-y PMC9487063

[27] Helmink BA , Reddy SM , Gao J , et al. B cells and tertiary lymphoid structures promote immunotherapy response. Nature. 2020;577(7791):549‐555. 31942075 10.1038/s41586-019-1922-8 PMC8762581

[28] Vanhersecke L , Brunet M , Guégan J‐P , et al. Mature tertiary lymphoid structures predict immune checkpoint inhibitor efficacy in solid tumors independently of PD‐L1 expression. Nat Cancer. 2021;2(8):794‐802. 35118423 10.1038/s43018-021-00232-6 PMC8809887

[29] Lynch KT , Young SJ , Meneveau MO , et al. Heterogeneity in tertiary lymphoid structure B‐cells correlates with patient survival in metastatic melanoma. J Immunother Cancer. 2021;9(6):e002273. 34103353 10.1136/jitc-2020-002273 PMC8190052

[30] Schreiber RD , Old LJ , Smyth MJ . Cancer immunoediting: integrating immunity's roles in cancer suppression and promotion. Science. 2011;331(6024):1565‐1570. 21436444 10.1126/science.1203486

[31] Gu‐Trantien C , Migliori E , Buisseret L , et al. CXCL13‐producing TFH cells link immune suppression and adaptive memory in human breast cancer. JCI Insight. 2017;2(11):e91487. 28570278 10.1172/jci.insight.91487 PMC5453706

[32] Kieffer Y , Hocine HR , Gentric G , et al. Single‐cell analysis reveals fibroblast clusters linked to immunotherapy resistance in cancer. Cancer Discov. 2020;10(9):1330‐1351. 32434947 10.1158/2159-8290.CD-19-1384

[33] Tie Y , Tang F , Wei Y‐Q , Wei X‐W . Immunosuppressive cells in cancer: mechanisms and potential therapeutic targets. J Hematol Oncol. 2022;15(1):61. 35585567 10.1186/s13045-022-01282-8 PMC9118588

[34] Desmedt C , Piette F , Loi S , et al. Strong time dependence of the 76‐gene prognostic signature for node‐negative breast cancer patients in the TRANSBIG multicenter independent validation series. Clin Cancer Res. 2007;13(11):3207‐3214. 17545524 10.1158/1078-0432.CCR-06-2765

[35] Sui Q , Zhang X , Chen C , et al. Inflammation promotes resistance to immune checkpoint inhibitors in high microsatellite instability colorectal cancer. Nat Commun. 2022;13(1):7316. 36443332 10.1038/s41467-022-35096-6 PMC9705377

[36] Valabrega G , Montemurro F , Aglietta M . Trastuzumab: mechanism of action, resistance and future perspectives in HER2‐overexpressing breast cancer. Ann Oncol. 2007;18(6):977‐984. 17229773 10.1093/annonc/mdl475

[37] Schütz F , Stefanovic S , Mayer L , von Au A , Domschke C , Sohn C . PD‐1/PD‐L1 pathway in breast cancer. Oncol Res Treat. 2017;40(5):294‐297. 28346916 10.1159/000464353

[38] Liu X , Xie P , Hao N , et al. HIF‐1‐regulated expression of calreticulin promotes breast tumorigenesis and progression through Wnt/β‐catenin pathway activation. Proc Natl Acad Sci U S A. 2021;118(44):e2109144118. 34706936 10.1073/pnas.2109144118 PMC8612225

[39] Jézéquel P , Loussouarn D , Guérin‐Charbonnel C , et al. Gene‐expression molecular subtyping of triple‐negative breast cancer tumours: importance of immune response. Breast Cancer Res. 2015;17:43. 25887482 10.1186/s13058-015-0550-y PMC4389408

[40] Zhang Y , Chen H , Mo H , et al. Single‐cell analyses reveal key immune cell subsets associated with response to PD‐L1 blockade in triple‐negative breast cancer. Cancer Cell. 2021;39(12):1578‐1593. 34653365 10.1016/j.ccell.2021.09.010

[41] Zhao H , Wang H , Kong F , et al. Oncolytic adenovirus rAd.DCN inhibits breast tumor growth and lung metastasis in an immune‐competent orthotopic xenograft model. Hum Gene Ther. 2019;30(2):197‐210. 30032645 10.1089/hum.2018.055

[42] Zhou Y , Luo G . Apolipoproteins, as the carrier proteins for lipids, are involved in the development of breast cancer. Clin Transl Oncol. 2020;22(11):1952‐1962. 32306242 10.1007/s12094-020-02354-2 PMC7505814

[43] Liu Y , Zhou Q , Song S , Tang S . Integrating metabolic reprogramming and metabolic imaging to predict breast cancer therapeutic responses. Trends Endocrinol Metab. 2021;32(10):762‐775. 34340886 10.1016/j.tem.2021.07.001

[44] Li Z , Zhang H . Reprogramming of glucose, fatty acid and amino acid metabolism for cancer progression. Cell Mol Life Sci. 2016;73(2):377‐392. 26499846 10.1007/s00018-015-2070-4 PMC11108301

[45] Liu X , Qiao Y , Ting X , Si W . Isocitrate dehydrogenase 3A, a rate‐limiting enzyme of the TCA cycle, promotes hepatocellular carcinoma migration and invasion through regulation of MTA1, a core component of the NuRD complex. Am J Cancer Res. 2020;10(10):3212‐3229. 33163266 PMC7642667

[46] Ulloa‐Montoya F , Louahed J , Dizier B , et al. Predictive gene signature in MAGE‐A3 antigen‐specific cancer immunotherapy. J Clin Oncol. 2013;31(19):2388‐2395. 23715562 10.1200/JCO.2012.44.3762

[47] Hatzis C , Pusztai L , Valero V , et al. A genomic predictor of response and survival following taxane‐anthracycline chemotherapy for invasive breast cancer. JAMA. 2011;305(18):1873‐1881. 21558518 10.1001/jama.2011.593 PMC5638042

[48] Necchi A , Joseph RW , Loriot Y , et al. Atezolizumab in platinum‐treated locally advanced or metastatic urothelial carcinoma: post‐progression outcomes from the phase II IMvigor210 study. Ann Oncol. 2017;28(12):3044‐3050. 28950298 10.1093/annonc/mdx518 PMC5834063

[49] Wouters MCA , Nelson BH . Prognostic significance of tumor‐infiltrating B cells and plasma cells in human cancer. Clin Cancer Res. 2018;24(24):6125‐6135. 30049748 10.1158/1078-0432.CCR-18-1481

[50] Franchina DG , Grusdat M , Brenner D . B‐cell metabolic remodeling and cancer. Trends Cancer. 2018;4(2):138‐150. 29458963 10.1016/j.trecan.2017.12.006

[51] Wang S‐S , Liu W , Ly D , Xu H , Qu L , Zhang L . Tumor‐infiltrating B cells: their role and application in anti‐tumor immunity in lung cancer. Cell Mol Immunol. 2019;16(1):6‐18. 29628498 10.1038/s41423-018-0027-x PMC6318290

[52] Gu Y , Liu Y , Fu L , et al. Tumor‐educated B cells selectively promote breast cancer lymph node metastasis by HSPA4‐targeting IgG. Nat Med. 2019;25(2):312‐322. 30643287 10.1038/s41591-018-0309-y

[53] Ding S , Chen X , Shen K . Single‐cell RNA sequencing in breast cancer: understanding tumor heterogeneity and paving roads to individualized therapy. Cancer Commun (Lond). 2020;40(8):329‐344. 32654419 10.1002/cac2.12078 PMC7427308

[54] Lin Z , Huang L , Li S , Gu J , Cui X , Zhou Y . Pan‐cancer analysis of genomic properties and clinical outcome associated with tumor tertiary lymphoid structure. Sci Rep. 2020;10(1):21530. 33299035 10.1038/s41598-020-78560-3 PMC7725838

[55] Li R , Berglund A , Zemp L , et al. The 12‐CK score: global measurement of tertiary lymphoid structures. Front Immunol. 2021;12:694079. 34267760 10.3389/fimmu.2021.694079 PMC8276102

[56] Zhao H , Wang H , Zhao Y , Sun Q , Ren X . Tumor‐resident T cells, associated with tertiary lymphoid structure maturity, improve survival in patients with stage III lung adenocarcinoma. Front Immunol. 2022;13:877689. 35663939 10.3389/fimmu.2022.877689 PMC9161276

[57] Castino GF , Cortese N , Capretti G , et al. Spatial distribution of B cells predicts prognosis in human pancreatic adenocarcinoma. Oncoimmunology. 2016;5(4):e1085147. 27141376 10.1080/2162402X.2015.1085147 PMC4839336

