# Supplementary figures and images for: Single‐cell transcriptome sequencing of B‐cell heterogeneity and tertiary lymphoid structure predicts breast cancer prognosis and neoadjuvant therapy efficacy
Source: Clin Transl Med. 2023 Aug 1;13(8):e1346. doi: 10.1002/ctm2.1346 (PMC10390819; doi:10.1002/ctm2.1346)

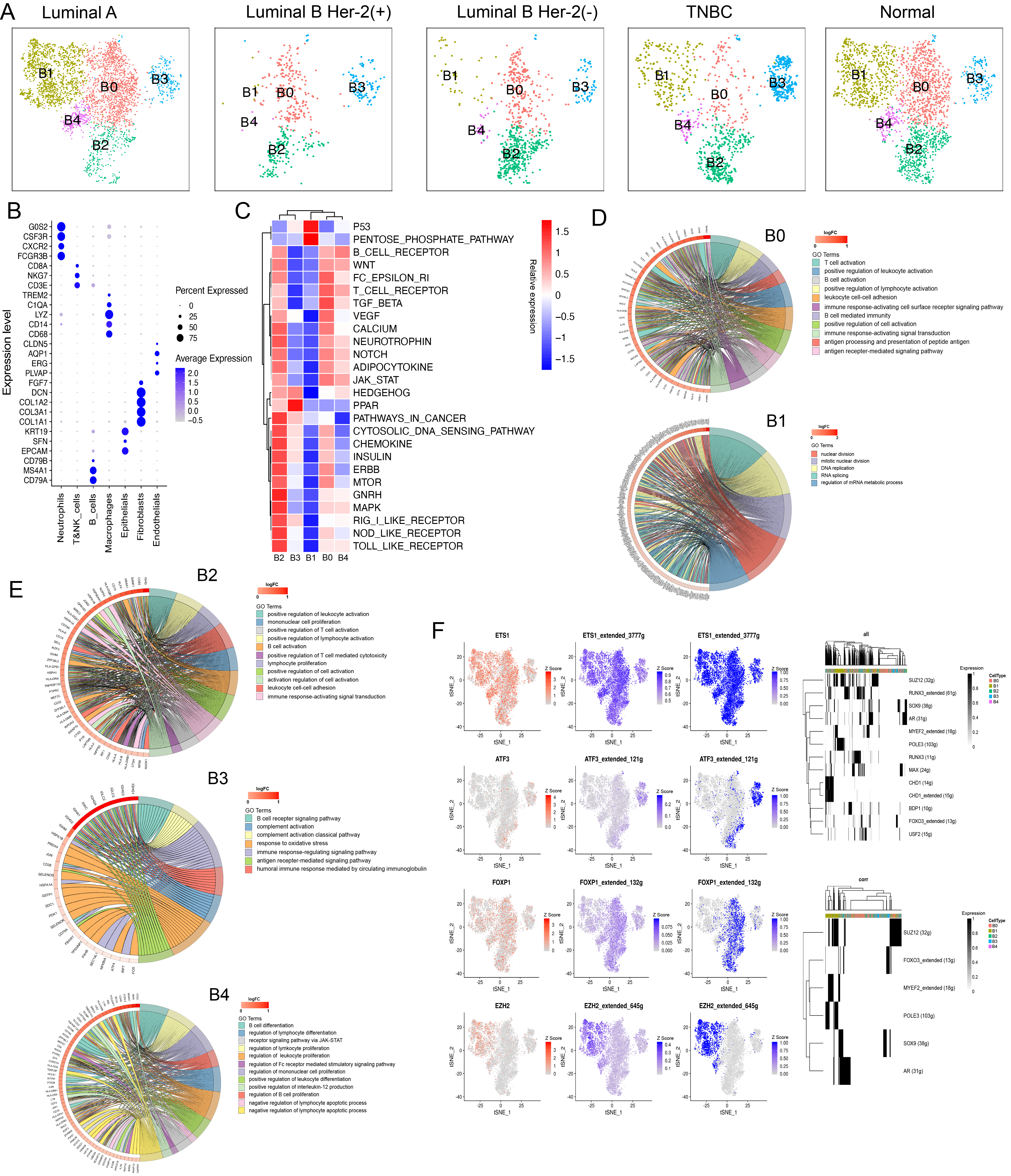

Supplement: Supplementary file 1 — (A) The tSNE plot illustrates the expression patterns of TIL‐B across distinct molecular subtypes within a cohort comprising 14 BC patients. (B) Utilizing multi‐marker genes, we employed Dotplot visualization to illustrate the diverse cellular types in 14 BC patients. (C) Pathway enrichment analysis based on the KEGG was conducted to investigate the TIL‐B subsets in 14 BC patients. (D and E) Diagram of chord illustrating the specific GO functional enrichment of each TIL‐B subgroup in 14 BC patients. (F) TIL‐B SENIC study in 14 BC patients. tSNE plot displaying the regulatory intensity of distinct transcription factors (TFs) in TIL‐B (left) and heat map displaying the regulatory intensity of TFs in TIL‐B (right). [file CTM2-13-e1346-s012.tif]

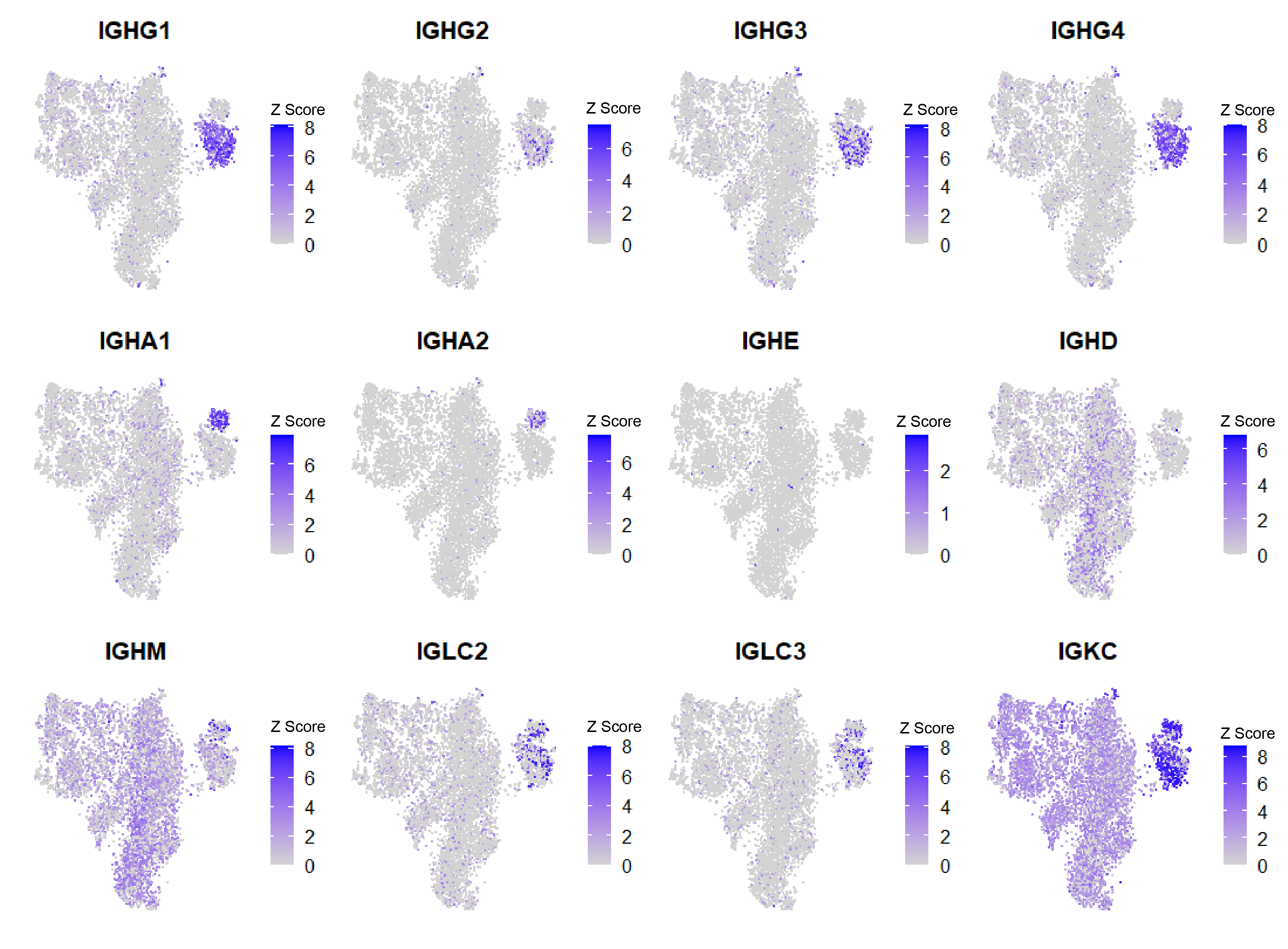

Supplement: Supplementary file 2 — Figure S2 Heavy and light chain expression in B cells as a whole. The UMAP projection reveals IGHG1, IGHG2, IGHG3, IGHG4, IGHA1, IGHA2, IGHE, IGHD, IGHM, IGLC2, IGLC3, and IGKC expression in TIL‐B within 14 BC patients. [file CTM2-13-e1346-s003.tif]

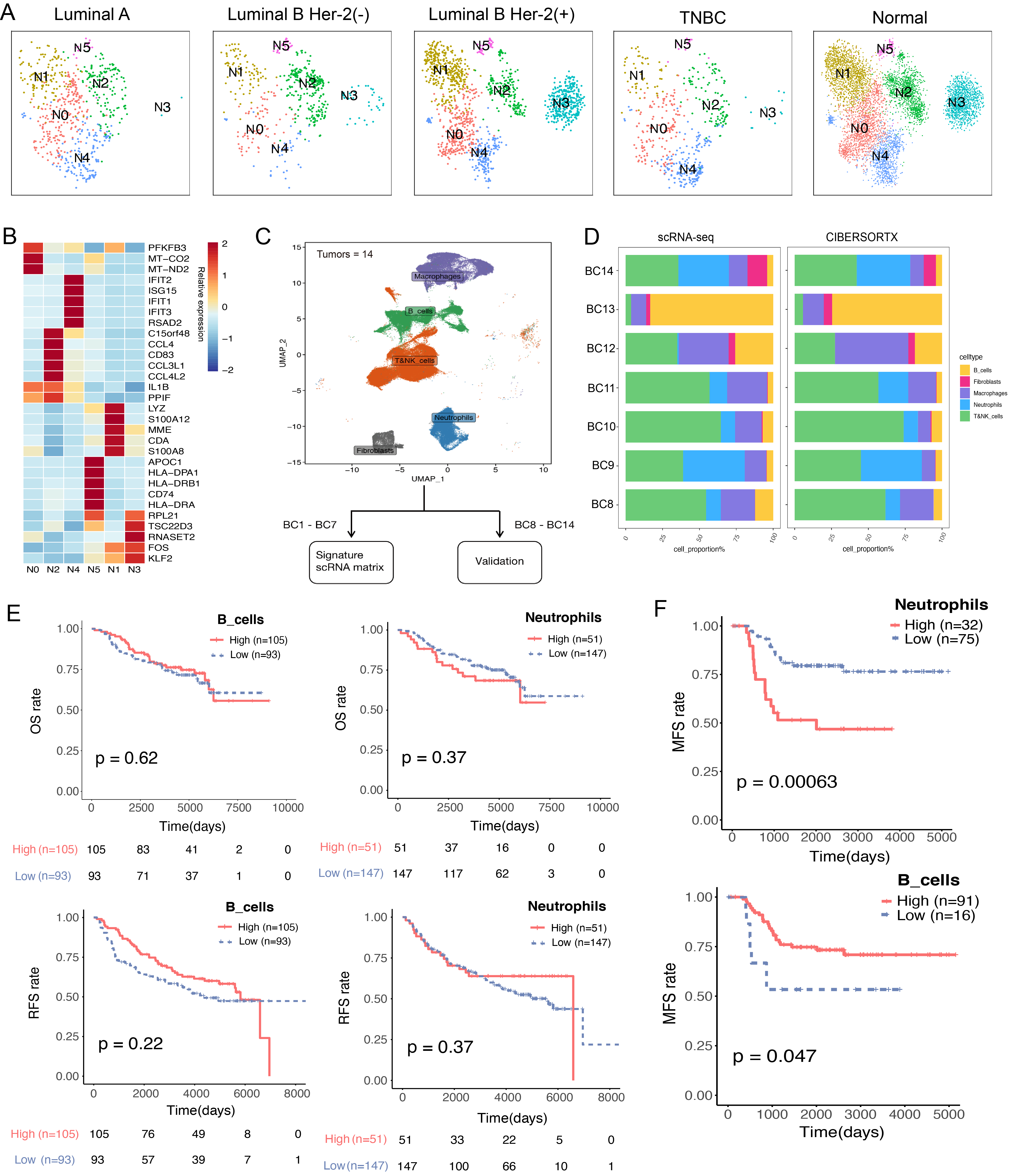

Supplement: Supplementary file 3 — Figure S3 Functional status of TAN subpopulations in BC. The tSNE plot illustrates the expression patterns of TAN across distinct molecular subtypes within 14 BC patients. (A) The TAN sub‐clusters' highly expressed genes in 14 BC patients are shown on the heatmap. (B) UMAP display of our scRNA‐seq data from 14 BC tissues (upper) and method for evaluating single‐cell deconvolution performance (bottom). (C) Concordance of cell type proportions of six major cell types as determined by CIBERSORTx deconvolution. (D) The Kaplan–Meier curve revealed that the degree of B cell and neutrophil infiltration was not significantly linked with OS and metastasis‐free survival (RFS)in the BC cohort (GSE7390). (E) The Kaplan–Meier curve revealed that in the TNBC cohort (GSE58812), patients with higher B cell infiltration had a better RFS (upper), while patients with higher neutrophil infiltration had a worse RFS (bottom). [file CTM2-13-e1346-s005.tif]

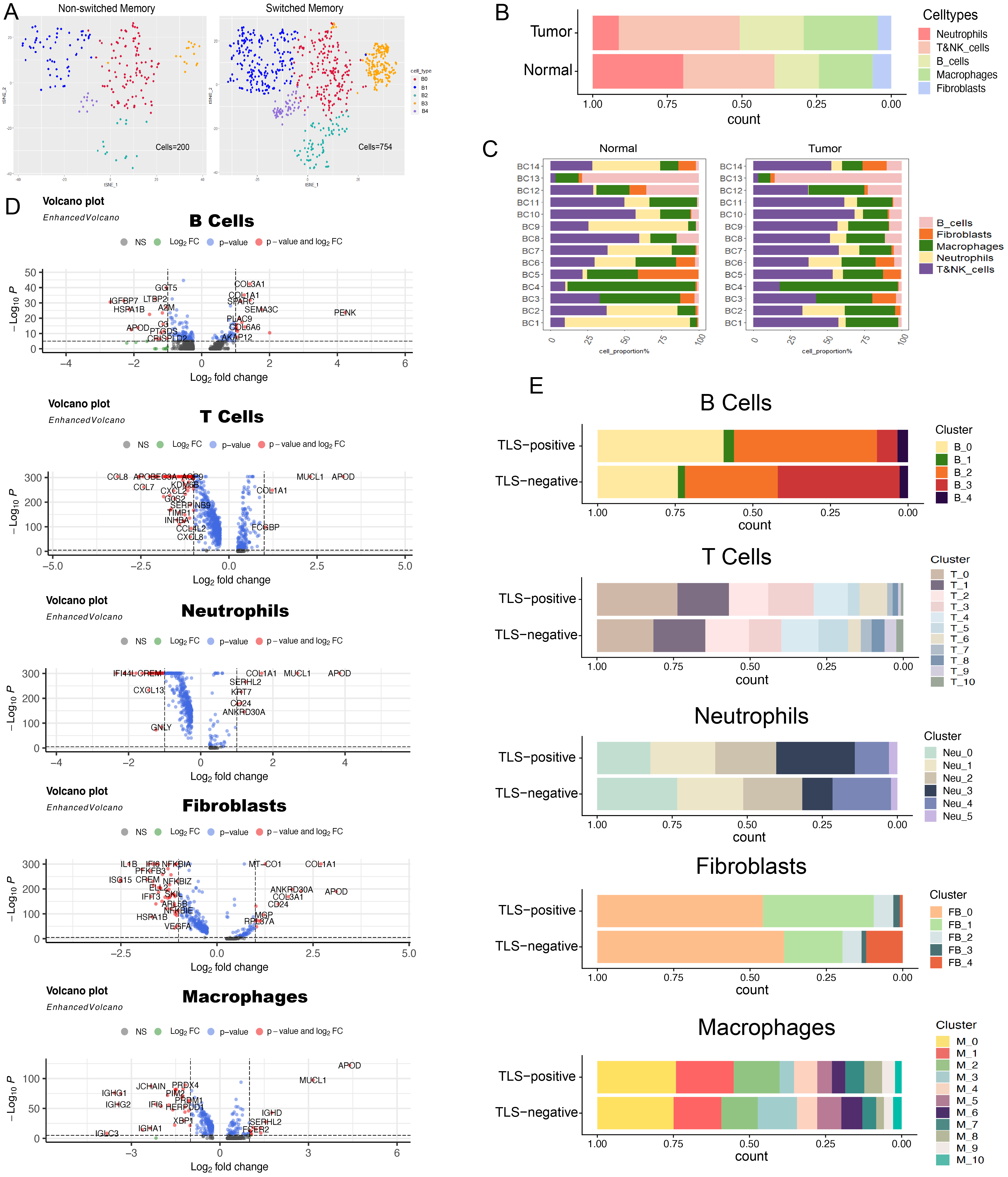

Supplement: Supplementary file 4 — Figure S4 Detailed characterization of Immune cells in BC. (A) The tSNE plot depicts the relative proportion of non‐switched memory B cells (left) and switched memory B cells (right) in 14 BC patients. (B) The compositions between normal and tumour tissues for immune cells in 14 BC patients. (C) The proportiions of immune cells between normal (left) and tumour (right) tissues in 14 BC patients. (D) DEGs analysis for each immune subsets in TLS‐positive and TLS‐negative groups of 9 BC patients. (E) The composition difference between TLS‐positive and TLS‐negative groups of 9 BC patients for each immune subsets. [file CTM2-13-e1346-s007.tif]

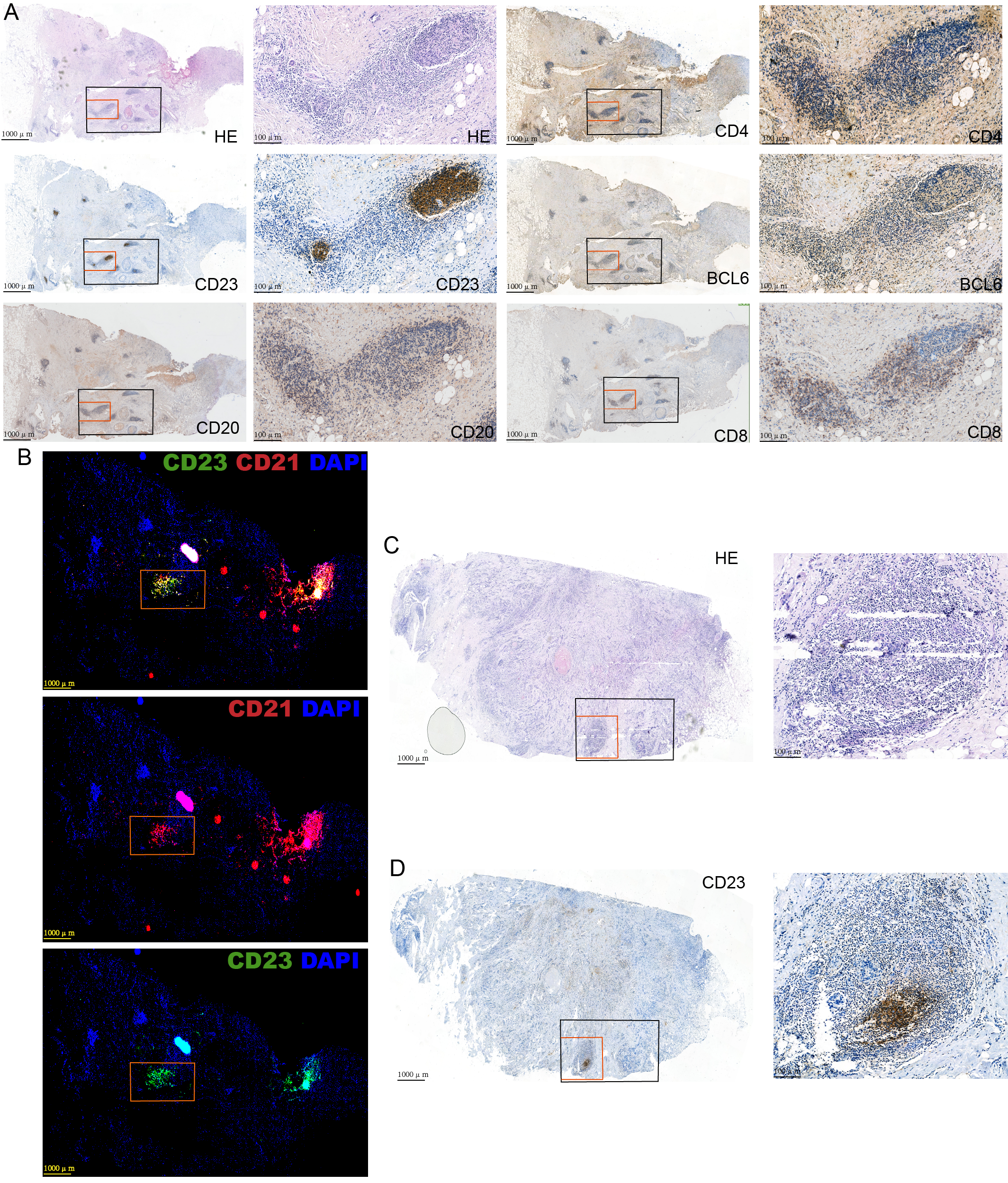

Supplement: Supplementary file 5 — Figure S5 Expression of TLSs and CD23 in BC. (A) Representative overview and details of H&E staining and IHC staining of CD4, CD8, CD20, CD23 and BCL6 expression in TLS‐positive BC patients. The images in the black rectangle box correspond to the images in Figure 5C, and the images in the red rectangle box correspond to the TLS details on their right. (B) Overview of CD23 and CD21 co‐immunofluorescence analysis in CD23‐positive TLS. The position of the orange rectangle corresponds to the image in Figure 5B. (C and D) Overview of H&E (C) and CD23 IHC (D) images of the typical TLS‐positive case. The images in the black rectangle box correspond to the images in Figure 5E‐5F, and the images in the red rectangle box correspond to the TLS details on their right. [file CTM2-13-e1346-s001.tif]

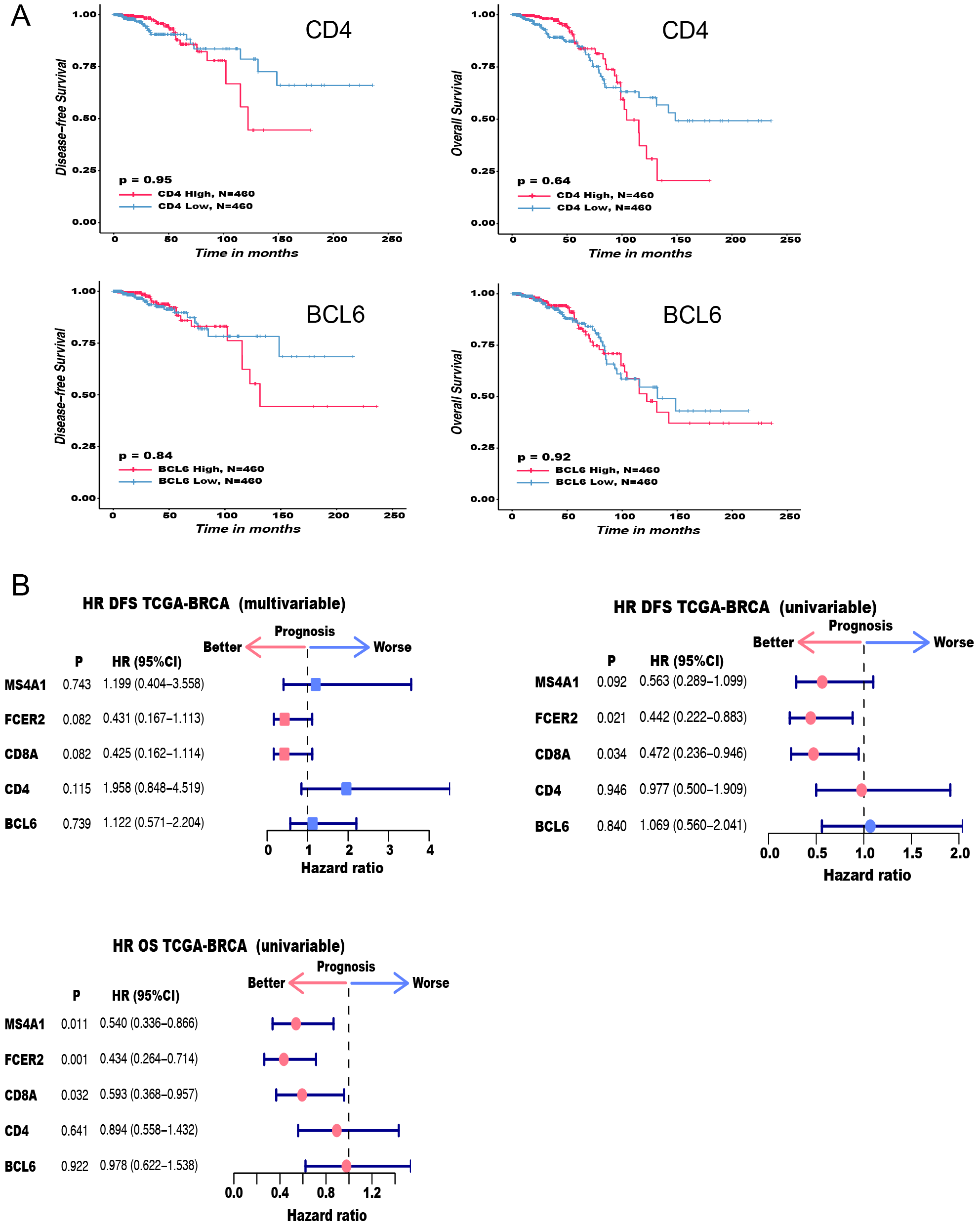

Supplement: Supplementary file 6 — Figure S6 Survival analysis of BRCA patients in the TCGA dataset. (A) Kaplan–Meier survival curves for the DFS (left panels) and OS (right panels) of 920 TCGA BRCA patients based on single gene expression (CD4 and BCL6). (B) The forest map shows HRs (center pink and blue squares and circles) and 95% confidence interval (horizontal ranges), and PSM matching has been made for factors such as molecular typing, lymph node status, tumour size, diagnosis age and histological grading of BC. [file CTM2-13-e1346-s002.tif]

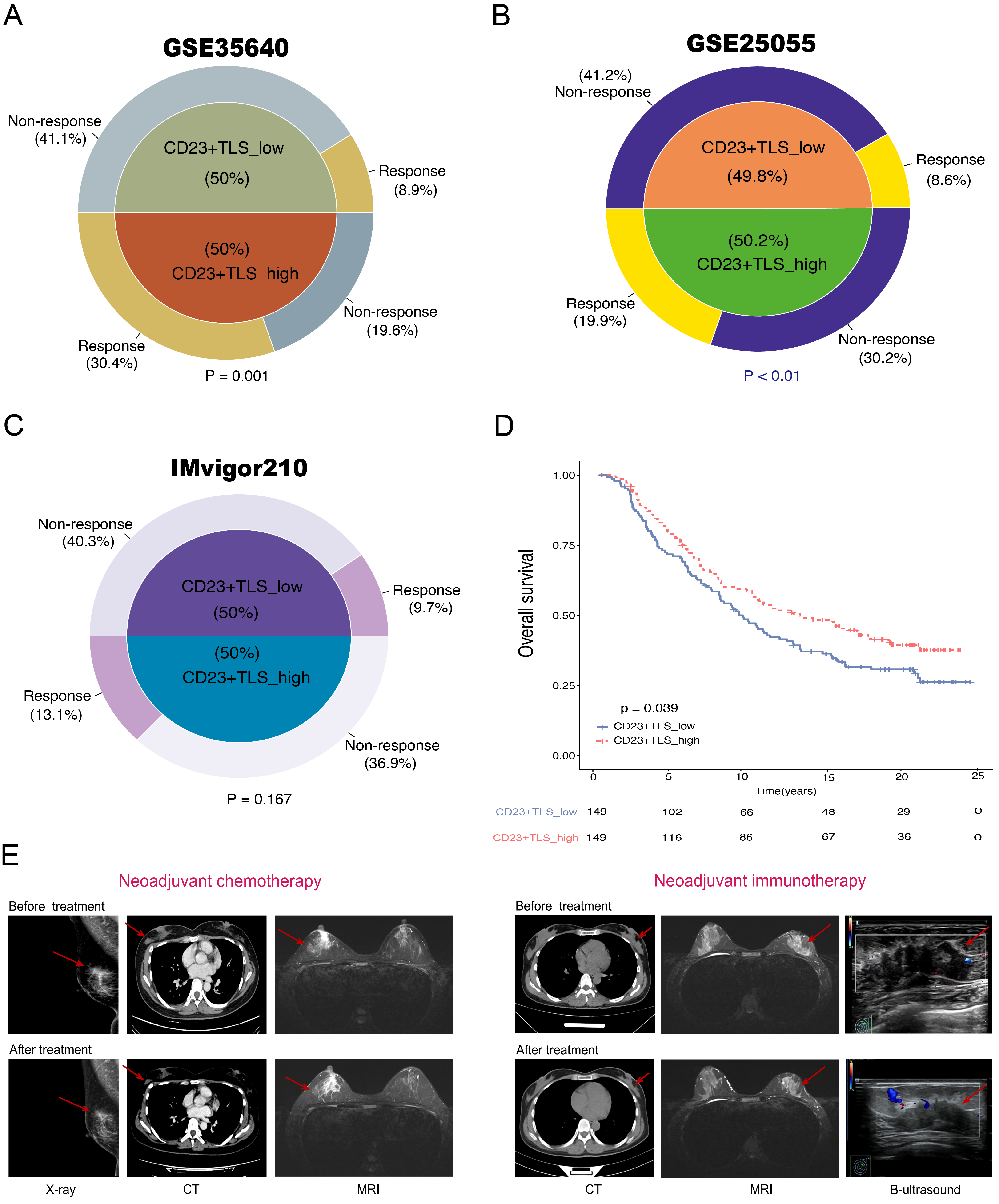

Supplement: Supplementary file 7 — Figure S7 The presence of mature TLS is associated with better patient survival and treatment response. (A–C) In three separate immunotherapy cohorts, GSE35640 (melanoma), GSE25055 (BC) and IMvigor210 (urothelial carcinoma), the association between TLS and treatment response is depicted by the circle diagram. (D) The presence of mTLS is related to better OS in IMvigor210, according to the Kaplan–Meier curve. (E) Imaging examinations demonstrate the therapeutic efficacy in TLS‐negative patients pre‐ and post‐administration of neoadjuvant chemotherapy (left) and neoadjuvant immunotherapy (right). [file CTM2-13-e1346-s006.tif]
