# Supplementary material for: Single‐cell transcriptome sequencing of B‐cell heterogeneity and tertiary lymphoid structure predicts breast cancer prognosis and neoadjuvant therapy efficacy
Source: Clin Transl Med. 2023 Aug 1;13(8):e1346. doi: 10.1002/ctm2.1346 (PMC10390819; doi:10.1002/ctm2.1346)
Supplement: Supplementary file 12 — Table S5 Clinical and pathological features of patients with and without TLS. [file CTM2-13-e1346-s004.docx]

**Table 5. Clinicopathological information of patients with neoadjuvant therapy**

| Characteristics | TLS-positive | | TLS-negative | |
| --- | --- | --- | --- | --- |
|  | Neoadjuvant chemotherapy | Neoadjuvant immunotherapy | Neoadjuvant chemotherapy | Neoadjuvant immunotherapy |
| Age | 37 | 42 | 46 | 69 |
| Sex | Female | Female | Female | Female |
| Histotype | IDC | IDC | IDC | IDC |
| ER（IHC） | 10% | - | - | - |
| PR（IHC） | 25% | - | 30% | - |
| HER-2（IHC） | - | - | ++（Fish-） | - |
| Ki-67（IHC） | 40% | 50% | 60% | 60% |
| Molecular typing | Luminal B | TNBC | Luminal B | TNBC |
| Axillary Node Status | - | 1/15 | 3/18 | - |
| TNM | cT2N0M0 | cT2N1M0 | cT3N1M0 | cT2N0M0 |
| Stage | IIA | IIB | IIIA | IIA |

ER: estrogen receptor; PR: progesterone receptor; HER-2: Human epidermal growth factor receptor 2
